# The buoyancy of cryptococcal cells and its implications for transport and persistence of *Cryptococcus* in aqueous environments

**DOI:** 10.1128/msphere.00848-24

**Published:** 2024-11-27

**Authors:** Isabel A. Jimenez, Piotr R. Stempinski, Quigly Dragotakes, Seth D. Greengo, Lia Sanchez Ramirez, Arturo Casadevall

**Affiliations:** 1 Department of Molecular and Comparative Pathobiology, The Johns Hopkins University School of Medicine 1500 , Baltimore, Maryland, USA; 2 Department of Molecular Microbiology and Immunology, Johns Hopkins University Bloomberg School of Public Health 25802 , Baltimore, Maryland, USA; University of Georgia, Athens, Georgia, USA

**Keywords:** environmental pathogens, halocline, pathogen transmission, marine mammals, public health, water, wildlife health

## Abstract

*Cryptococcus* is a genus of saprophytic fungi with global distribution. Two species complexes, *Cryptococcus neoformans* and *Cryptococcus gattii*, pose health risks to humans and animals. Cryptococcal infections result from inhalation of aerosolized spores and/or desiccated yeasts from terrestrial reservoirs such as soil and trees. More recently, *C. gattii* has been implicated in infections in marine mammals, suggesting that inhalation of cells from the air-water interface is also an important, yet understudied, mode of respiratory exposure. Based on historical records and epidemiological factors, water transport has been hypothesized to play a role in the spread of *C. gattii* from tropical to temperate environments. However, the dynamics of fungal persistence and transport in water have not been fully studied. The size of the cryptococcal capsule was previously shown to reduce cell density and increase buoyancy. Here, we demonstrate that cell buoyancy is also impacted by the salinity of the solution in which cells are suspended, with the formation of a halocline significantly slowing the rate of settling and resulting in persistence of *C. neoformans* within 1 cm of the water surface for over 60 min and *C. gattii* for 4–6 h. During the culture of three strains of *C. gattii* in yeast peptone dextrose media, we also identified aggregates of extracellular polysaccharide with complex structures, which we hypothesize from rafts that entrap cells and augment buoyancy. These findings illustrate new mechanisms by which cryptococcal cells may persist in aquatic environments, with important implications for aqueous transport and pathogen exposure.

**IMPORTANCE:**

Cryptococcosis is a major fungal disease leading to morbidity and mortality worldwide. *Cryptococcus neoformans* is a major fungal species of public health concern, causing opportunistic systemic infections in immunocompromised patients. *Cryptococcus gattii* was traditionally a pathogenic fungus confined primarily to tropical regions, but in the 1990s, it emerged in the temperate climates of British Columbia, Canada and the Pacific Northwest of the United States. Outbreaks in these areas also led to the first host record of cryptococcosis in free-ranging cetaceans. *C. gattii* is particularly concerning as an emerging fungal pathogen due to its capacity to cause clinical disease in immunocompetent patients, its recent spread to a new ecological niche, and its higher resistance to antifungal therapies. Our research defines fungal characteristics that influence the transport of cryptococci through water and persistence of fungal cells near the water surface, improving our understanding of potential mechanisms for cryptococcal environmental transport.

## INTRODUCTION

*Cryptococcus* is a genus of environmental fungi with global distribution. Two members of this genus, *Cryptococcus neoformans* and *Cryptococcus gattii*, belong to species complexes that cause pulmonary and neurological infections in humans, domestic animals, and wildlife (1–7). *C. neoformans* has a worldwide distribution and is ubiquitous in the environment, classically found in dirt and avian guano (8). *C. gattii* is predominantly found in association with trees and was formerly geographically restricted to tropical and subtropical regions, but since the 1990s, it has emerged as the cause of outbreaks in humans and animals in the temperate regions of British Columbia, Canada and the Pacific Northwest (PNW) of the United States (9), raising new questions regarding the epidemiology of this pathogen (7, 10). Multiple studies have linked *C. gattii* isolates in British Columbia and the PNW to origins in Brazil (11–14), with molecular clock analyses suggesting that translocation occurred approximately 100 years ago (13). *C. gattii* has since been widely isolated from air, soil, water, and trees in this region, with human and environmental isolates predominantly identified from coastal areas (9).

Two models explain how *C. gattii* reached this new ecological niche: land-first and ocean-first (12, 15). Given the natural association of *C. gattii* with trees, anthropogenic transport of lumber or fomites such as logging equipment or vehicle tires could potentially result in sporadic contamination of land sites with *C. gattii*. However, no land-first hypotheses have been proposed to account for the current degree of extensive contamination of PNW forests nor for a mechanism of subsequent widespread dissemination of cryptococcal cells from contaminated land sites into coastal waters (15). Engelthaler et al. (13) proposed an ocean-first hypothesis in which, following the 1914 opening of the Panama Canal, cargo ships transported *C. gattii* to North America via contaminated ballast water (13). Engelthaler and Casadevall (15) subsequently proposed that *C. gattii* cells surviving in coastal waters were later washed into nearby forests by flooding of land due to a 1964 tsunami, resulting in large-scale contamination of the forests in which *C. gattii* is now considered endemic (15). The capacity for aqueous transport raises the possibility that cryptococcal species could spread via ocean currents. However, the hypothesis that water plays a key role in cryptococcal dispersal and propagation relies on two key assumptions: (i) that cells can survive for prolonged periods of time in water; and (ii) that cells are capable of remaining buoyant long enough to be transported large distances in water.

Experimentally, *C*. *gattii* survived for up to 1 year in both ocean water and freshwater (9). Multiple cryptococcal species have been identified in water samples, including *C. gattii* (9, 16, 17), *C. neoformans* (18), *C. albidus*, *C. laurentii*, and *C. humicolus* (19–21). Overall, *Cryptococcus* spp. have been identified across fresh, brackish, and ocean water, from coasts to deep sea trenches, at water surfaces, and from biofilms in municipal water systems (9, 16–29). *C. gattii* outbreaks in the PNW also resulted in the first cases of cryptococcosis in free-ranging marine mammals (1, 10, 30); in fact, a 1997 case of pulmonary cryptococcosis in a Dall’s porpoise (*Phocoenoides dalli*) in Washington state preceded the first regional human case in 1999 (10).

Cryptococcal infections classically involve inhalation of dry aerosols in the form of spores and/or desiccated yeasts from terrestrial environmental reservoirs, such as soil, wood, dust, and dried avian guano (31–33). The emergence of marine mammal cryptococcal cases suggests that these aerosols generated on land could be carried out over the water by wind currents and inhaled near the water surface (10, 15). We further propose that cryptococcal cells that land on the water surface or are washed into the ocean at estuaries may remain suspended in water, then become aerosolized and inhaled when marine mammals surface to breathe. This model would indicate that liquid droplets and wet aerosols may present an additional understudied mode of respiratory exposure. Defining factors that influence the persistence of cryptococci in aquatic environments is therefore pertinent to understanding not only the ocean-first hypothesis but also disease transmission to susceptible humans and animals.

A major virulence factor of *Cryptococcus* is the capsule, composed of branched polysaccharides anchored at the cell wall and radiating outward with decreasing density (34, 35). Prior work from our laboratory demonstrates that larger capsules decrease cell density and thus increase buoyancy, potentially serving as a flotation device and facilitating dispersion through water (36). In the current study, we further analyzed the contribution of the capsule to buoyancy. In addition, because cryptococci in soils have small capsules (37), we hypothesized that the capsule may not be the primary mechanism by which cryptococcal cells remain buoyant when washed from land to sea. We sought to evaluate additional mechanisms by which cryptococci could persist in water, with a particular focus on persistence near the water surface. Here, we report that cryptococci utilize a variety of mechanisms to remain suspended in water, and aquatic environments can support prolonged buoyancy of cryptococcal cells.

## MATERIALS AND METHODS

### Yeast strains, culture conditions, and media

Frozen stocks of *C. neoformans* (serotype A reference strain H99 [genotype VNI; ATCC 208821] and acapsular *cap59* deletion mutant [serotype D strain C536, derived from B-3501 parental strain]; [38, 39]) and *C. gattii* (environmental isolate WM161 [genotype VGIII; ATCC MYA-4562], feline clinical isolate NIH409 [40] [genotype VGIII], and Vancouver Island clinical isolate R265 [5] [genotype VGIIa]) were inoculated into liquid yeast peptone dextrose (YPD) media (BD Difco, Sparks, MD) and incubated in a culture rotator (37 rpm) at 30°C for 48 h. Stationary phase cultures were streaked onto solid YPD media (BD Difco), incubated at 30°C for 48 h and stored at 4°C for up to 14 d until inoculation into liquid culture. Unless otherwise indicated, all strains were utilized in each experiment, and cells were cultured in liquid YPD media at 30°C until stationary phase. Minimal media (MM) was prepared as previously described (41). Pacific Ocean seawater (SW) (Imagitarium, Petco, San Diego, CA) and live Nutri-Seawater Aquarium Saltwater (LSW) (Nature’s Ocean, Fort Lauderdale, FL) were purchased from commercial vendors; macro- and trace-element compositions are shown in Table S1. Where indicated, SW was filter-sterilized using a 0.22 µM filter (MilliporeSigma, Burlington, MA).

### Cell imaging and measurements

Cells were photographed with India ink counterstaining on an Olympus AX70 microscope (Olympus America, Melville, NY) at 20× or 40× magnification, with a QImaging Retiga 1300 camera using QCapture software (QImaging, Burnaby, British Columbia, Canada) (42). Cell diameter and cell body diameter were measured from at least 50 cells per condition in Fiji (43), and capsule volume was calculated (42). Capsule:body volume ratio was calculated by dividing capsule volume by cell body volume.

### Density gradient centrifugation

Cells were washed and resuspended in phosphate-buffered saline (PBS). A working solution of Percoll (MilliporeSigma) density gradient medium was prepared to a final osmolality of 1.0914 g/mL, as previously described (36). Colored polyethylene Density Marker Beads (DMBs; Cospheric, Santa Barbara, CA) were used as density standards (green, 1.02 g/cc; orange, 1.04 g/cc; violet, 1.06 g/cc; dark blue, 1.08 g/cc; red, 1.09 g/cc; medium blue, 1.13 g/cc). A volume of 50 µL (1 × 10^7^ cells) of each culture or 20 µL of each DMB was added to 13 × 51 mm polypropylene centrifuge tubes (Beckman Coulter, Sykesville, MD) containing 3 mL of Working Percoll Solution (WPS). Tubes were balanced with WPS and centrifuged using an Optima TLX tabletop ultracentrifuge (Beckman Coulter) with TLA 100.3 fixed angle rotor at 40,000 rpm at 25°C for 30 min (36). Tubes were photographed using a Nikon D3000 DSLR camera under uniform positioning and light conditions. The “Plot Profile” function in Fiji was used to measure the grayscale intensity of each pixel along a vertical line of 800 pixels in length, drawn through an equivalent section of each image. Values for each strain were normalized by subtracting the average background value to account for effects of minor differences in lighting on grayscale intensity. Grayscale intensity was plotted as a function of distance using Graphpad Prism 10.1.1.

### Antibody collection and purification/modification

Murine monoclonal antibodies (mAbs) used for this study were IgG1 18B7 (44) (Unisyn Technologies) and IgM 12A1 (45). IgM 12A1 was purified from ascites supernatants using a HiTrap IgM Purification HP (Cytiva) according to the manufacturer protocol. The concentrations of the purified antibodies were determined using a Pierce BCA Protein Assay Kit (Thermo Fisher Scientific, Waltham, MA) according to the manufacturer’s protocol. For a subset of experiments, 18B7 was directly conjugated to fluorophore Oregon Green 488 (18B7-OG) using an Oregon Green TM 488 Protein Labeling Kit (Thermo Fisher Scientific) according to the manufacturer’s protocol.

### Capture enzyme-linked immunosorbent assay (ELISA)

Capture ELISAs were performed using a modified version of a previously described protocol (46). IgM 12A1 and IgG1 18B7 were selected for the capture ELISA due to their ability to broadly bind glucuronoxylomannan (GXM) glycans (47). Each well in a 96-well flat-bottomed plate was coated with 1 µg/mL unlabeled goat-anti-mouse IgM (SouthernBioTech, Birmingham, AL) in PBS and incubated for 1 h at 37°C. The plate was removed from incubation and blocking solution [1% bovine serum albumin (BSA) in PBS] was added, and then the plate was incubated for 1 h at 37°C. Well contents were removed, 2 µg/mL IgM 12A1 in blocking solution was added to each well, and the plate was incubated for 1 h at 37°C. Wells were washed three times with Tris-buffered saline with 0.1% Tween-20 detergent (TBST) (MilliporeSigma) with a MultiWash+ microplate washer (Molecular Devices, San Jose, CA). A dilution curve of H99 whole exopolysaccharide (EPS), supernatant samples diluted in blocking solution, and blocking solution negative controls were added in duplicate to the plate and incubated at 4°C overnight. After incubation, plates were washed as before, and 5 µg/mL IgG1 18B7 in blocking solution was added to each well. Plates were incubated for 1 h at 37°C, then emptied and washed as before. A solution of 1 µg/mL goat anti-mouse IgG (SouthernBioTech) in blocking buffer was added to the wells, and then plates were incubated for 1 h at 37°C. Plates were washed, and 1 mg/mL p-nitrophenyl phosphate (MilliporeSigma) in ELISA substrate buffer (1 mM MgCl_2_·6 H_2_O, 50 mM Na_2_CO_3_, and pH 9.8 in deionized [DI] H_2_O) was added to each well and allowed to develop at room temperature. Absorbances at *λ* = 405 nm were measured using a SpectraMax i5 plate reader (Molecular Diagnostics). Data were normalized to the average reading for plain YPD media, and then analyzed and plotted using GraphPad Prism 10.1.1.

### Immunofluorescence imaging

Samples (50 µL) were washed once, pelleted, and resuspended in 200 µL of a 10 µg/mL solution of IgG1 18B7 in 1% BSA-PBS blocking buffer to label GXM. Samples were incubated at 4°C overnight with gentle agitation. Cells were washed and incubated at room temperature for 1 h with 2.5 µg/mL goat anti-mouse IgG Alexa-Fluor 488 secondary antibody (Thermo Fisher Scientific) and 5 µg/mL Uvitex 2B (Polysciences Inc., Warrington, PA) to label cell wall chitin, in 1% BSA-HBSS. In a separate set of experiments, to image polysaccharide rafts, stationary phase cultures of each strain were allowed to passively settle until a translucent upper layer was visible, at which time the upper layer was collected (H99, 1.5 h; WM161, 20 h; NIH409, 26.5 h; R265, 26.5 h). For *cap59*, no upper layer was visible; therefore, a sample was taken after 45 min. To preserve polysaccharide architecture, no washes were performed. Samples were incubated at room temperature for 1 h with 5 µg/mL of Uvitex 2B and 10 µg/mL of 18B7-OG and then imaged on a Leica THUNDER Live Cell and 3D Confocal Microscope at 63× (oil objective). Minimum and maximum brightness of each image channel were set uniformly for all images, and composites were created in Fiji.

### Focused ion beam scanning electron microscopy

Scanning electron microscopy (SEM) was used to image samples collected from a stationary phase culture of strain NIH409 (0 h timepoint) and samples collected from the translucent upper layer after 16 h of passive settling (16 h timepoint). Samples were fixed in 2.5% glutaraldehyde, 3 mM MgCl_2_, in 0.05 M sodium cacodylate buffer, pH 7.2 overnight at 4°C. After buffer rinse, samples were postfixed in 1% osmium tetroxide in 0.075 M sodium cacodylate buffer on ice in the dark for 1 h. Following a DH_2_O rinse, samples were dehydrated in a graded series of ethanol and left to dry overnight in a desiccator with hexamethyldisilazane. Samples were mounted on carbon-coated stubs and imaged on a JEOL Field Emission SEM (JSM-IT700HR InTouchScope) at 2 kV.

### Capsule size after 3 d incubation in different media types

Baseline cell measurements were taken from stationary phase cultures in liquid YPD media. As a positive control, 50 µL of culture was inoculated into 5 mL of MM and incubated for 3 d to induce capsular enlargement. Remaining culture samples were separated into two 5 mL aliquots per strain, centrifuged at 2,300 g for 4 min, washed twice in PBS, and then resuspended in PBS or filter-sterilized SW before incubation for 3 d. Cell measurements were repeated.

### Effect of acute saltwater incubation on capsule size

To assess for acute physicochemical effects of sodium chloride (NaCl) on capsule size, cells of strain R265 were cultured overnight in YPD, washed once in DI water, and then incubated in DI water for 1 h to dialyze the capsules against hypotonic media, which has previously been shown to induce expansion of the capsular gel (48). Subsequently, 500 µL aliquots of cells in DI water were incubated for 1 h in each of the following conditions: DI water, 0.2 M NaCl, 1.5 M NaCl, 5 M NaCl, and filtered SW. Cell measurements were taken using immunofluorescence imaging. Samples were incubated for 45 min with 5 µg/mL of Uvitex 2B and 5 µg/mL of 18B7-OG with gentle agitation, and then, 2 µL of samples was added onto a 1% agarose patch and imaged at 63× on an Olympus AX70 microscope. Cell measurements were obtained.

### Halocline formation

Halocline interfaces form in nature whenever fresh water flows over salt water, resulting in vertical stratification of the fluids by density, with low-density fresh water forming a relatively stable surface layer. To demonstrate halocline formation as a function of differences in specific gravity (SG) between layers, phenol-red (PR) indicator (MilliporeSigma) was dissolved in PBS or SW, and a volume of 200 µL of each solution (“suspension media”) was added to cuvettes containing 3 mL of either PBS, SW, or LSW (“cuvette media”). Cuvettes were photographed at intervals. In a separate experiment, a standard curve of NaCl was prepared (Table S2), and 3 mL of each solution was added to cuvettes. An overnight YPD culture of H99 was washed once in PBS, cells were resuspended in a solution of PBS with phenol-red indicator dye (PBS-PR), and 200 µL of cell suspension was added to each cuvette. Photographs were taken within 1 min. To test the impact of the halocline layer on cell settling while keeping salinity constant, cells of strain R265 in 200 µL of suspension media with either 0.0, 1.5, or 5.5 M [NaCl] were added to 3 mL of cuvette media with either 1.6, 1.5, or 1.25 M [NaCl], respectively, such that the final [NaCl] in each cuvette would be 1.5 M. Cuvettes were photographed at intervals between 5 min and 6 h. To demonstrate the dynamic persistence of the halocline layer, cells of strain R265 were suspended in PBS-PR, and this cell suspension was layered onto SW in a culture flask and placed on a horizontal plate shaker, incrementally increasing the number of rotations per min, while video was captured (Video S1).

### Buoyancy and settling assays

To evaluate the rate of passive settling of cells in YPD culture, cells at a final concentration of 2–3 × 10^8^ cells/mL and final volume of 3 mL were added to cuvettes and allowed to passively settle. Cuvettes were photographed at intervals between 5 min and 26.5 h. To assess the impact of salinity on the rate of cell settling, cuvettes were prepared with 3 mL of PBS or filtered SW. Cells were washed once, and 1–3 × 10^7^ cells were resuspended in 200 µL of PBS or filtered SW and then gently added to the top of each cuvette. Control cuvettes received 200 µL of PBS-PR. Cuvettes were photographed at intervals between 5 min and 6 h.

### Polysaccharide dilution curve

To evaluate the influence of the amount of extracellular GXM on the rate of cell settling, a serial dilution of strain NIH409 and R265 cultures was performed, in which 3, 2.25, 1.5, and 0.75 mL of the original cultures were supplemented with 0, 0.75, 1.5, or 2.25 mL of washed cells in fresh YPD media, respectively (Fig. S1). This effectively diluted out extracellular contents of the culture while keeping the cell count constant. Cuvettes were photographed at intervals between 5 min and 10 h. The concentration of GXM from each sample was measured by capture ELISA to confirm that these methods resulted in serial dilution of GXM.

### Settling rate calculation

A cuvette was marked in millimeter intervals and photographed under identical conditions to experimental cuvettes. Adobe Photoshop was used to add digital measurement lines, and the distance between the water surface and the upper border of suspended cells was measured. Cell settling was calculated as the rate of displacement from the surface over time.

### Phenol-sulfuric acid assay

An overnight culture of strain NIH409 was allowed to passively settle for approximately 18 h at room temperature before the collection of 500 µL of the translucent upper layer. Concurrently, a 500 µL sample of stationary phase overnight culture was collected. Samples were diluted (1:100), vortexed to disrupt large polysaccharide aggregates, and centrifuged at 2,300 g for 4 min. The supernatant was saved, and the pellet was washed twice and resuspended in water. A phenol-sulfuric acid (PSA) assay to detect total polysaccharides was performed as previously described (49). Absorbance was measured at 490 nm, and readings were normalized to background readings from control wells of water. Polysaccharide concentration (µg/mL) was calculated using the standard curve and normalized to the cell count of each sample.

### Enrichment of GXM content by passive settling

Cells of strains H99, NIH409, R265, and WM161 were cultured in YPD and then allowed to passively settle for 2 or 6 h before the collection of 500 µL of upper and lower layers. Samples were plated at 1:100,000 dilution on solid YPD agar and incubated for 2 d at 30°C to determine colony-forming units (CFUs). Each sample was also vortexed at maximum speed for 1 min and centrifuged to pellet the cells, and the supernatant was collected for GXM ELISA.

### Lipid quantification by fluorescence microscopy and lipid droplet induction

Cells of strains H99 and NIH409 were cultured in plain YPD media to determine baseline lipid content or cultured in YPD supplemented with 4 mM oleic acid (MilliporeSigma) to induce lipid droplet formation. To quantify lipid, culture samples (200 µL) were incubated for 5 min with 5 µg/mL Uvitex 2B, then incubated with 25 µL of 1:1 dimethylsulfoxide:PBS for 1 min to permeabilize cells, followed by 125 µg/mL of Nile Red in acetone for 5 min to stain neutral lipids (50, 51). Samples were imaged at 63× (oil objective). Nile Red fluorescence intensity was measured from at least 50 cells per strain and condition using Fiji. Readings were normalized to background fluorescence intensity from blank spaces. Culture samples were added to cuvettes and allowed to passively settle to determine the rate of settling.

### Specific gravity by refractometry

SG is the ratio between the density of a compound and the density of pure water at 4°C (1.000 g/cm^3^) and is proportional to the salinity of a liquid. SG of each media type was measured using a salinity refractometer, which was calibrated using DI water before each set of measurements.

### Inductively coupled plasma optical emission spectroscopy (ICP-OES)

Samples of SW and LSW were submitted to Triton Labs (Düsseldorf, Germany) for macro- and trace-element analysis by ICP-OES. Results are shown in Table S1.

### Statistical analysis

Statistical analyses were performed using GraphPad Prism 10.1.1. To assess for differences in the rate of settling, strains and conditions were compared by simple linear regression or nonlinear regression using a one-phase decay model, as indicated. To evaluate the significance of differences in capsule size, cell body size, and capsule:body volume ratio, a Kruskal-Wallis test with Dunn’s multiple comparisons testing was performed. To compare fluorescence intensity of samples stained with Nile Red, outliers were identified via robust regression and outlier removal (ROUT) (*Q* = 1%) and excluded from analysis, and unpaired *t*-tests were used to compare groups. Polysaccharide concentrations were compared using two-way analysis of variance (ANOVA).

## RESULTS

### Cell density and cell dimensions of five cryptococcal strains

**Fig 1:**
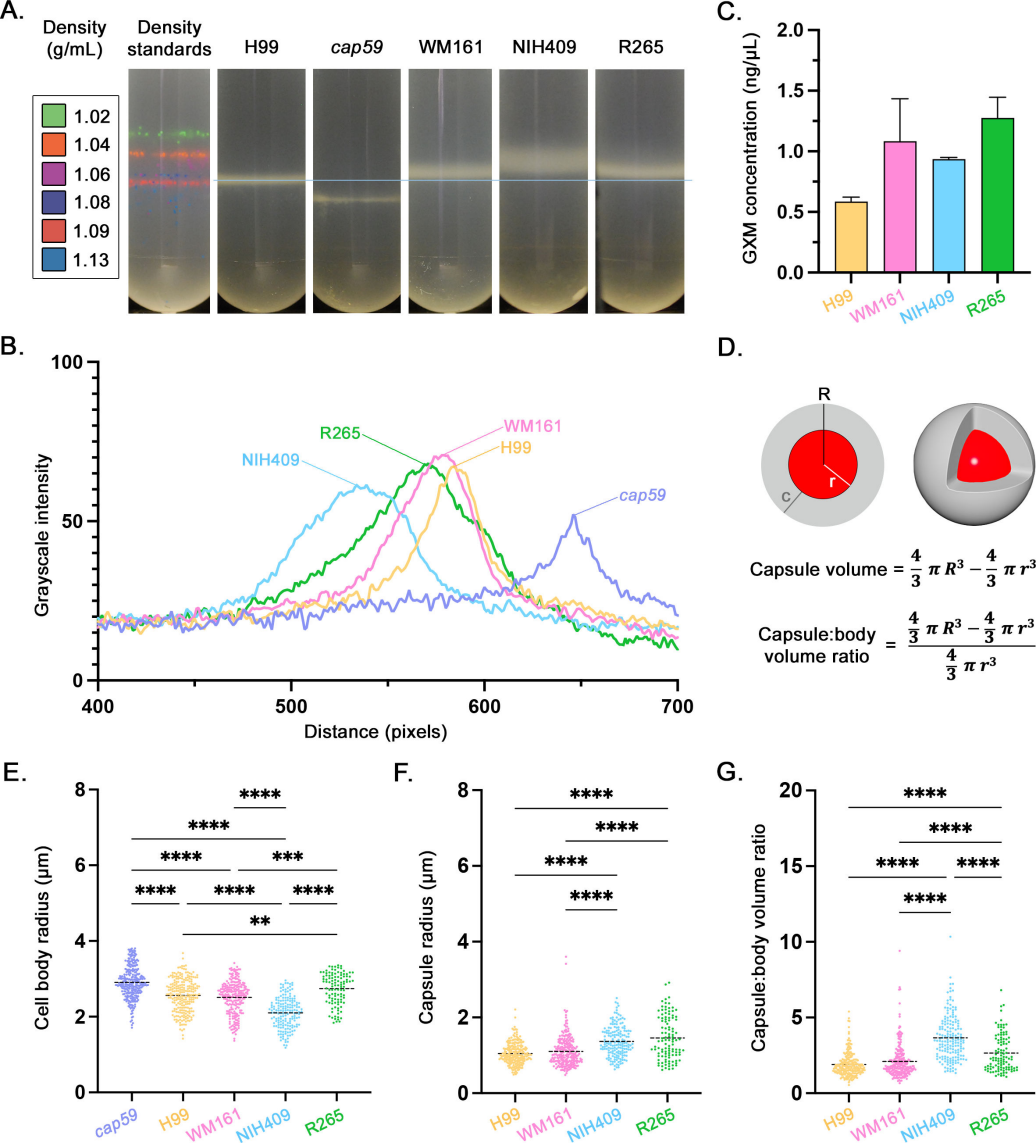
Comparison of cell density, extracellular GXM, and cell measurements of five strains of *Cryptococcus*. *C. neoformans* strains H99 and acapsular mutant *cap59*, and *C. gattii* strains WM161, NIH409, and R265, were cultured overnight in YPD media. (**A**) Cell density was evaluated using density gradient centrifugation, in comparison to a standard of DMBs. Strain *cap59* had the highest cell density (1.13 g/mL), followed by H99 (1.08–1.09 g/mL), WM161 (1.06–1.08), R265 (1.06–1.08), and NIH409 (1.03–1.07 g/mL). Results represent two independent experiments. (**B**) Grayscale intensity values per pixel were measured from a vertical line through each image and plotted against distance for each strain; the width of each peak represents the width of the band within the Percoll density medium, and increasing distance corresponds to increasing cell density. (**C**) Baseline concentration of extracellular GXM was measured for each strain by a capture ELISA, revealing no significant differences between strains. Results represent two independent experiments. (**D**) Diagram of a cryptococcal cell, with cell body (red) and capsule (gray). Measurements were taken of total radius (***R***), cell radius (***r***), and capsule radius (***c***). The capsule volume was calculated by subtracting the volume of the cell body from the volume of the entire cell. The ratio between capsule volume and cell body volume was calculated by dividing the capsule volume by the cell body volume. Results represent at least three independent experiments per strain, with at least 50 cells measured per replicate. ****: *P* < 0.0001; ***: *P* ≤ 0.001; **: *P* ≤ 0.01; *: *P* ≤ 0.05. (**E**) Cell body size varied significantly between strains. (**F**) Both NIH409 and R265 had significantly larger capsule radii compared to H99 and WM161. (**G**) Strain NIH409 had a significantly larger capsule:body volume ratio than H99, WM161, and R265, while R265 had a significantly larger capsule:body volume ratio than H99 and WM161.

We measured cell densities of five strains (H99, *cap59*, WM161, NIH409, and R265) using density gradient centrifugation and found differences in cell density and density heterogeneity, represented by differing band heights and widths, respectively, visible in the Percoll density medium (Fig. 1A). We then measured the grayscale intensity values per pixel within a vertical line through the center of each tube, generating a graph in which the width of each peak represents the width of the band within the Percoll density medium, and increasing distance corresponds to increasing cell density (Fig. 1B). These results demonstrate that *cap59* had the highest cell density and least variability of the strain evaluated, with progressively decreasing cell density for strains H99, WM161, R265, and NIH409. Baseline GXM concentration (ng/µL) was measured via ELISA, and no differences were present between strains (Fig. 1C). Cell body radius and capsule radius were measured, and capsule:body volume ratio was calculated (Fig. 1D). Strain-specific differences were present in cell body size (Fig. 1E) and capsule size (Fig. 1F). There were also marked differences in capsule:body volume ratio between strains (Fig. 1G) which aligned with the species-specific differences in cell density. Strain NIH409 had a larger capsule:body volume ratio than all other strains (*P* < 0.0001), and strain R265 had a larger capsule:body volume ratio than strains WM161 and H99 (*P* < 0.0001).

### Effect of aqueous culture conditions on capsule size

**Fig 2:**
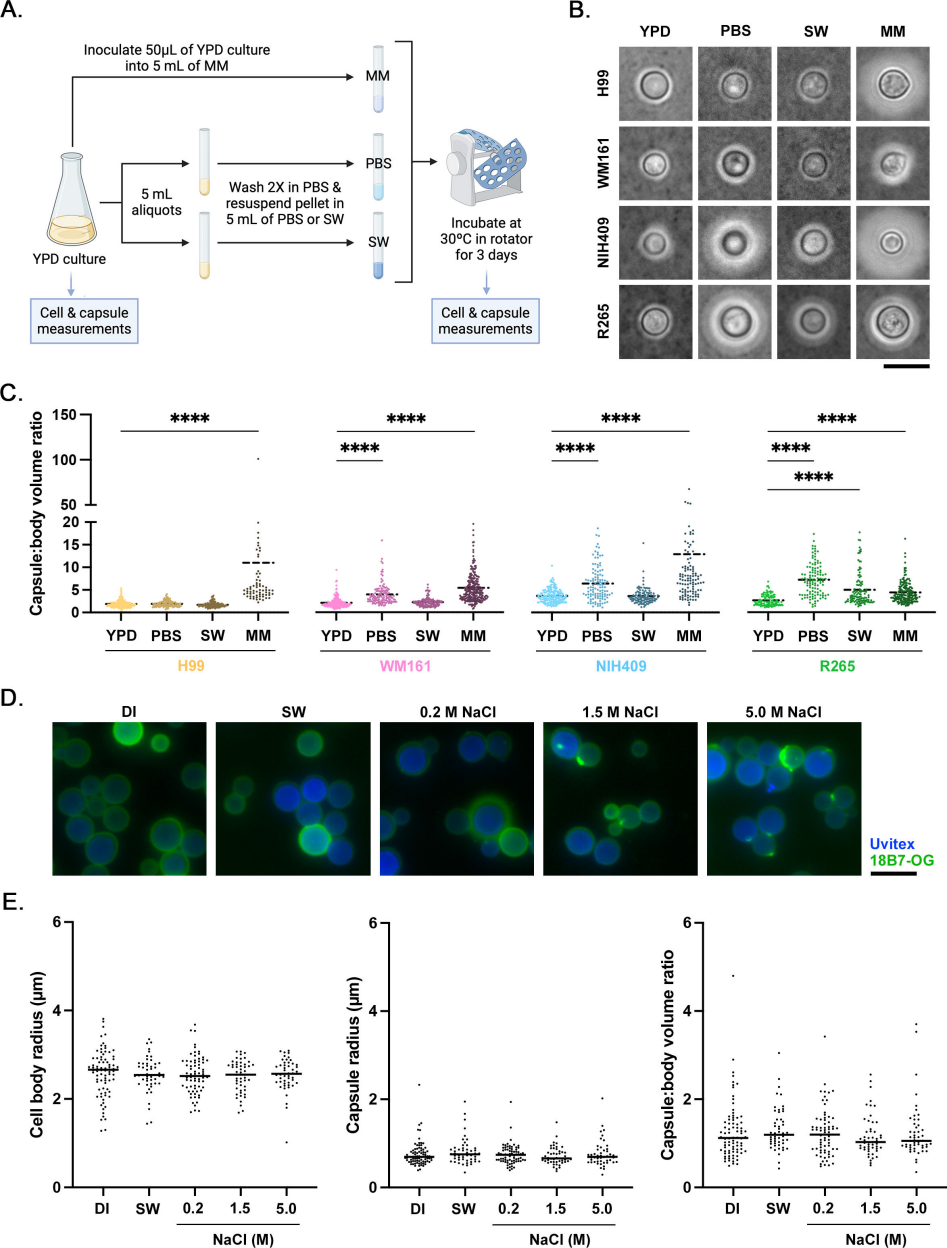
Capsule size after incubation in different media types. (**A**) To assess the effect of incubation in water on capsule growth, *C. neoformans* strains H99 and *C. gattii* strains NIH409, WM161, and R265 were cultured in YPD media, washed once in PBS, and then incubated for 3 d in PBS, filtered SW, or MM. (**B**) Microscopy was performed with India ink counterstaining to capture images of cells; shown here are representative images of relative differences in capsule size between strains and media conditions. Scale = 10 µm. (**C**) Capsules and cell bodies were measured, and capsule:body volume ratio was calculated. Results represent at least two independent experiments per strain and media condition, with at least 50 cells measured per replicate. ****: *P* < 0.0001; ***: *P* ≤ 0.001; **: *P* ≤ 0.01; *: *P* ≤ 0.05. The capsule:body volume ratio of strain R265 was significantly larger after incubation in SW (*P* < 0.0001), but this effect was not present in any other strain (*P* > 0.9999). All three *C. gattii* strains had larger average capsule:body volume ratios after PBS incubation than baseline (*P* < 0.0001), while the average capsule:body volume ratio of strain H99 did not change after PBS incubation (*P* > 0.9999). As expected, incubation in MM resulted in capsular growth in all strains (*P* < 0.0001). (**D**) To evaluate acute physicochemical effects of sodium chloride (NaCl) on capsule size, cells of strain R265 were cultured overnight in YPD media, washed and incubated in DI water for 1 h, and then incubated for 1 h in water with varying concentrations of NaCl. Cells were imaged using immunofluorescence microscopy, and cell measurements were obtained. (**E**) No significant differences in cell body radius, capsule radius, or capsule:body volume ratio were present between strains or conditions.

We next evaluated capsule size after incubation in different media types. Cells of four encapsulated strains were cultured in YPD media, washed, and then inoculated into MM, PBS, or filtered SW and incubated for 3 d (Fig. 2A). Light microscopy with India ink counterstaining was performed (Fig. 2B), and cell measurements were taken. As expected, MM incubation induced significant capsule enlargement in all strains compared to baseline YPD culture (*P* < 0.0001). Incubation in PBS resulted in a significant increase in capsule size for all three *C. gattii* strains (*P* < 0.0001) but not for *C. neoformans* strain H99 (*P* > 0.9999). Interestingly, a significant increase in capsule:body volume ratio was also observed following SW incubation of strain R265 (*P* < 0.0001), while no differences were seen for other strains (*P* > 0.9999; Fig. 2C). Thus, both high and low salinity water appeared to promote capsular growth in *C. gattii*.

### Effect of NaCl on capsular size

Prior studies have found that *C. neoformans* cells incubated in hyperosmolar conditions, including NaCl, had reduced capsule size (48, 52), and monovalent ions can induce GXM disaggregation (53, 54). To evaluate potential roles of NaCl in mediating capsular shedding or inducing physicochemical contraction of the capsule, we incubated *C. gattii* R265 cells in DI water and then dialyzed the cells against media of different concentrations of NaCl, modeled on work performed by Jacobson et al. (48). We then imaged cells by immunofluorescence microscopy (Fig. 2D). We found no effect of NaCl concentration on cell body radius, capsule radius, or capsule:body volume ratio (Fig. 2E), suggesting that SW does not induce physicochemical contraction or shedding of the capsule in YPD-grown *C. gattii* strain R265.

### Specific gravity by refractometry

SG of each media type was measured by salinity refractometry (Table 1). SG of cell suspensions, each at a concentration of 1 × 10^8^ cells/mL in each media type, were also measured; the addition of cells increased the SG by 0.000–0.002 compared to plain media.

**TABLE 1:** SG of media by salinity refractometry

Media	SG
Deionized water	1.000
MM	1.004
PBS, Ca^2+^ and Mg^2+^ free	1.006
PR + PBS	1.007
Pacific Ocean SW	1.026
PR + SW	1.026
LSW	1.030
YPD	1.035

### Experimental halocline interface formation

**Fig 3:**
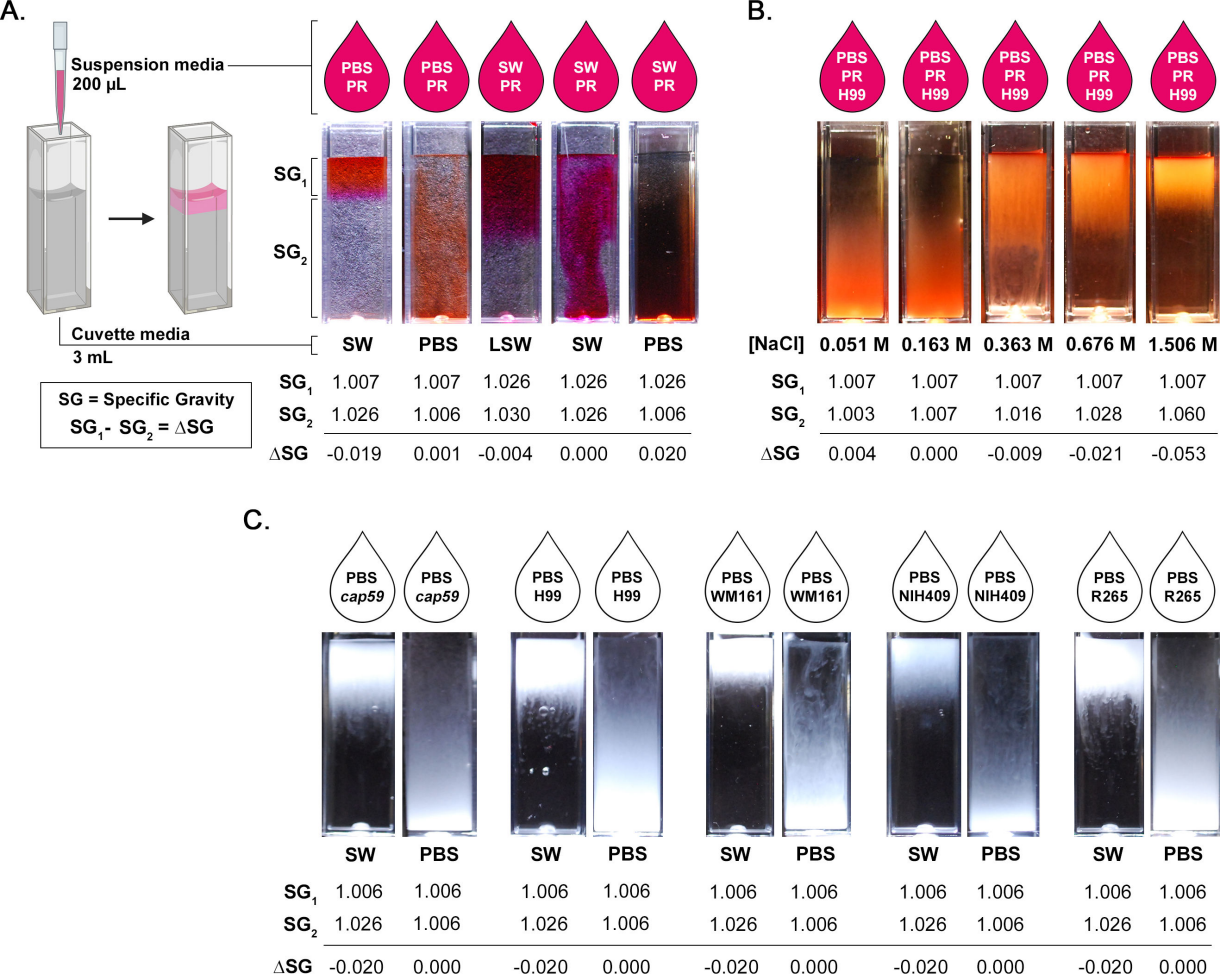
Experimental halocline formation results in suspension of cryptococci near the water surface. The SG of a liquid is the ratio between the density of the liquid and the density of pure water at 4°C (1.000 g/cm^3^) and is proportional to salinity. When two liquids are layered, the difference in SG (∆SG) determines if layers vertically stratify to form a halocline interface. (**A**) To demonstrate halocline interface formation, we filled cuvettes with 3 mL of liquid (“cuvette media”) and then gently added 200 µL of liquid (“suspension media”) colored with PR indicator dye to the top of each cuvette. ∆SG was calculated by subtracting the SG of the cuvette media from the SG of the suspension media. When ∆SG < 0, a halocline forms, as illustrated by the addition of PBS-PR onto a column of Pacific Ocean SW. Even very small differences in SG result in halocline formation, as shown by the addition of SW-PR (SG = 1.028) to SW from a different source (LSW; SG = 1.030). Conversely, when ∆SG ≥ 0, no halocline forms, and the liquids rapidly mix. (**B**) To illustrate the linear relationship between ∆SG and the size of the halocline, cells of strain H99 were suspended in PBS-PR and added to the top of cuvettes containing serial concentrations of sodium chloride (NaCl). (**C**) To demonstrate that halocline formation persists in the presence of cells and influences the suspension of those cells, cells of strains *cap59*, H99, WM161, NIH409, and R265 were suspended in PBS and then gently added to the top of cuvettes containing either PBS or SW. Photographs were taken within 1 min. When cells were suspended in PBS and added to SW, a halocline interface formed, with cells visible near the water surface. This is seen even in the acapsular *cap59* mutant, demonstrating that this effect is independent of capsule size. Conversely, when cells grown under identical conditions were suspended in PBS and added to PBS, the cells dispersed rapidly, demonstrating the marked impact of halocline layer formation on buoyancy.

We experimentally formed a halocline interface, with and without the presence of cryptococci, by filling cuvettes with 3 mL of various liquids (“cuvette media”) and gently adding 200 µL of other liquids (“suspension media”) to the surface (Fig. 3A). When the difference in SG (∆SG) between the suspension media (SG_1_) and the cuvette media (SG_2_) was negative, a halocline formed, as illustrated by addition of PBS-PR onto SW. Conversely, when ∆SG ≥ 0, the liquids rapidly mixed. Even very small differences in SG are important, as demonstrated by halocline formation after the addition of SW-PR (SG = 1.026) atop LSW (SG = 1.030; ∆SG = −0.004), while no halocline formed after the addition of PBS-PR (SG = 1.007) to a column of PBS (SG = 1.006; ∆SG = 0.001; Fig. 3A). To evaluate the influence of ∆SG on halocline size, we prepared a series of NaCl concentrations and added cells of strain H99, suspended in PBS-PR. As ∆SG became more negative, the halocline interface became narrower, and cells were visible closer to the water surface (Fig. 3B). To demonstrate that halocline formation was sufficient to suspend cryptococci near the water surface regardless of capsule size, we cultured cells of five strains in YPD media, suspended cells in PBS, and added the cell suspension on top of columns of PBS or filtered SW. For all strains, cells suspended in PBS were buoyant when added to a column of SW, but the same cell suspension sank rapidly when layered onto PBS (Fig. 3C). This pattern was conserved in the *cap59* acapsular mutant, confirming that this effect is independent of the presence of the capsule. We further illustrated that the halocline layer remained stable under dynamic conditions by layering R265 cells in PBS-PR on top of SW and gradually increasing the horizontal agitation (Video S1), with halocline layer persistence up to 100 rpm.

### The rate of cryptococcal cell settling varies inversely with salinity and the presence of a halocline

**Fig 4:**
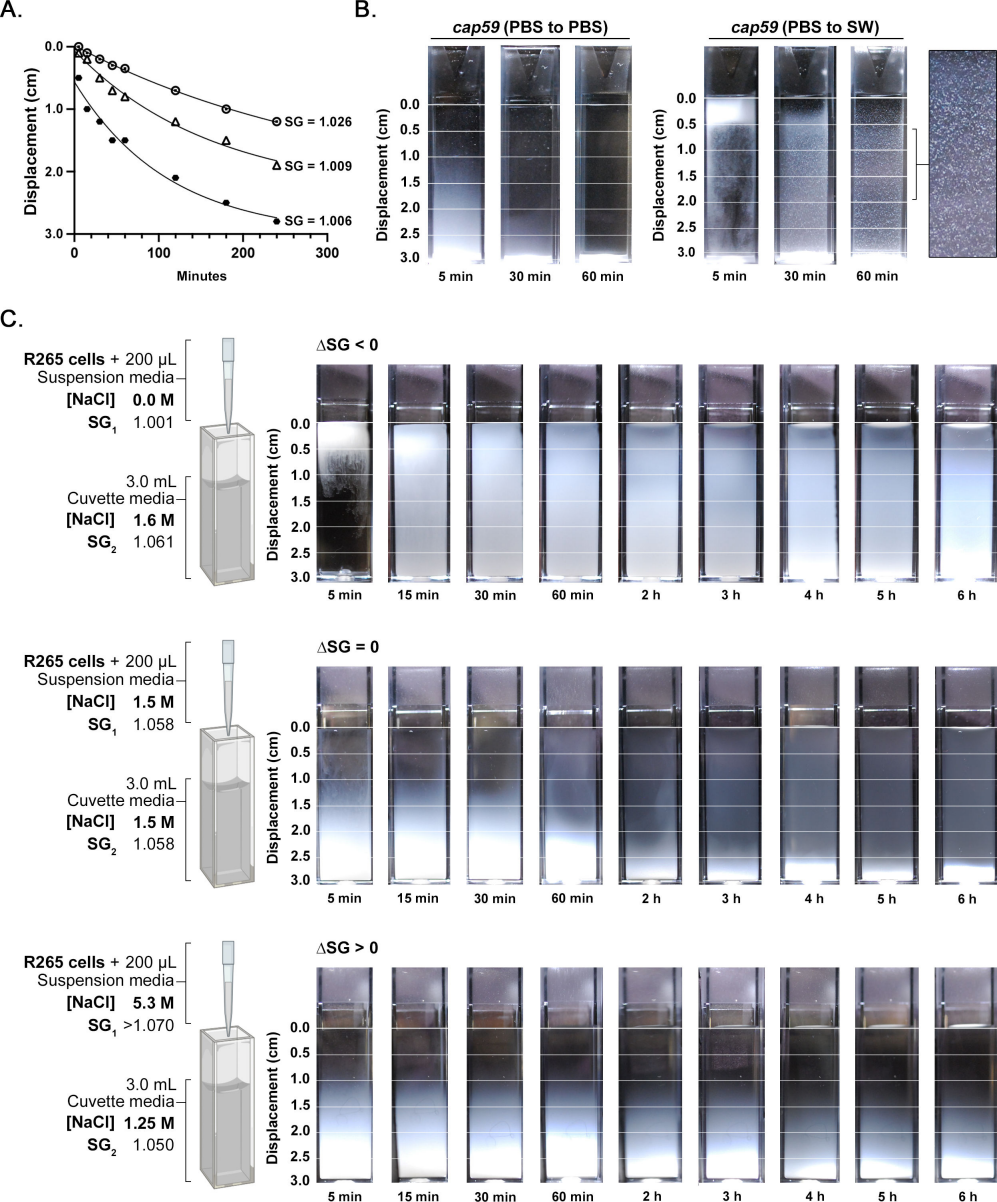
Salinity and halocline formation are both factors that delay settling of cryptococci in SW. (**A**) The rate at which cells settle through water is inversely proportional to the final SG of the water. Cells of strains H99, *cap59*, WM161, NIH409, and R265 were suspended in 200 µL of PBS or SW and added to the top of cuvettes containing 3 mL of either PBS or SW. Cells suspended in PBS and added to SW were initially suspended at the halocline interface and then moved out of the halocline over time. The rate of cell settling was assessed over 4 h by measuring displacement (cm) from the top of the cuvette. Media type significantly impacted the rate of cell settling (*P* < 0.0001). Cells suspended in PBS and added to PBS (final SG = 1.006) settled the fastest, while cells suspended in PBS and added to SW (final SG = 1.026) settled the slowest. The graph shows the settling rate of strain H99 under these three conditions; images are shown in Fig. S2. This finding was conserved among the other two strains tested (NIH409 and WM161) with images and graphs shown in Fig. S2. (**B**) Acapsular *cap59* cells suspended in PBS and added to SW exhibited marked clumping and adherence to the side of the cuvette, while *cap59* cells suspended in PBS and added to PBS did not exhibit clumping. (**C**) To specifically test the impact of the halocline layer on cell settling while avoiding confounding effects of salinity differences, we designed an experiment in which the final SG in each cuvette would equal 1.5 M NaCl. Cells of strain R265 were suspended in 200 µL of suspension media and then added to cuvettes such that the difference in SG (∆SG) would either induce halocline formation (∆SG < 0) or not (∆SG = 0 or ∆SG > 0). This experiment demonstrates that when all other factors are kept constant, the rate of cell settling is significantly slower in the presence of a halocline interface (*P* < 0.0001).

A buoyancy assay time course was performed from 5 min to 4 h in strains H99, WM161, NIH409, and R265 using PBS and SW. These media types were selected because the predominant SW used in this study is natural Pacific Ocean SW that matches the expected salinity and composition of ocean water. Cells suspended in 200 µL of PBS or SW were layered onto cuvettes containing 3 mL PBS or SW, photographs were taken at intervals, and the rate of cell settling was calculated. The rate of settling varied inversely with salinity, with a higher final SG in the cuvette corresponding to significantly slower settling based on nonlinear regression (Fig. 4A). This is consistent with the principle that higher salinity water is denser, and thus contributes more buoyant force, than low salinity water. However, another factor with an impact on buoyancy was involved in this experiment: when cells were suspended in PBS and added to a cuvette containing SW, a halocline layer formed. In the presence of a halocline, cells of strains H99, WM161, and NIH409 remained visible within the upper 1 cm of the cuvette for over 60 min (Fig. S2). Interestingly, we also noted that under the two combinations that incorporated SW, cells of acapsular mutant strain *cap59* exhibited marked macroscopic clumping and adherence to the walls of the cuvette (Fig. 4B).

To study the effect of the halocline layer on cell settling independent of the effects of salinity, we designed an experiment in which the individual salinities of the suspension media and cuvette media were titrated to maintain a constant final salinity of [NaCl] = 1.5 M across all conditions. Thus, we were able to assess the rate of settling of R265 cells in the presence of a halocline (ΔSG < 0) and compared this to the rate of settling under two conditions in which no halocline forms (ΔSG = 0 and ΔSG > 0), with all other factors kept constant. In this context, the rate of cell settling was significantly slower in the presence of a halocline (*P* < 0.0001). Taken together, these two experiments demonstrate that halocline layer formation significantly slows the rate of settling of cryptococcal cells in water, and this occurs at environmentally relevant NaCl concentrations.

### Strain-specific rates of passive settling

**Fig 5:**
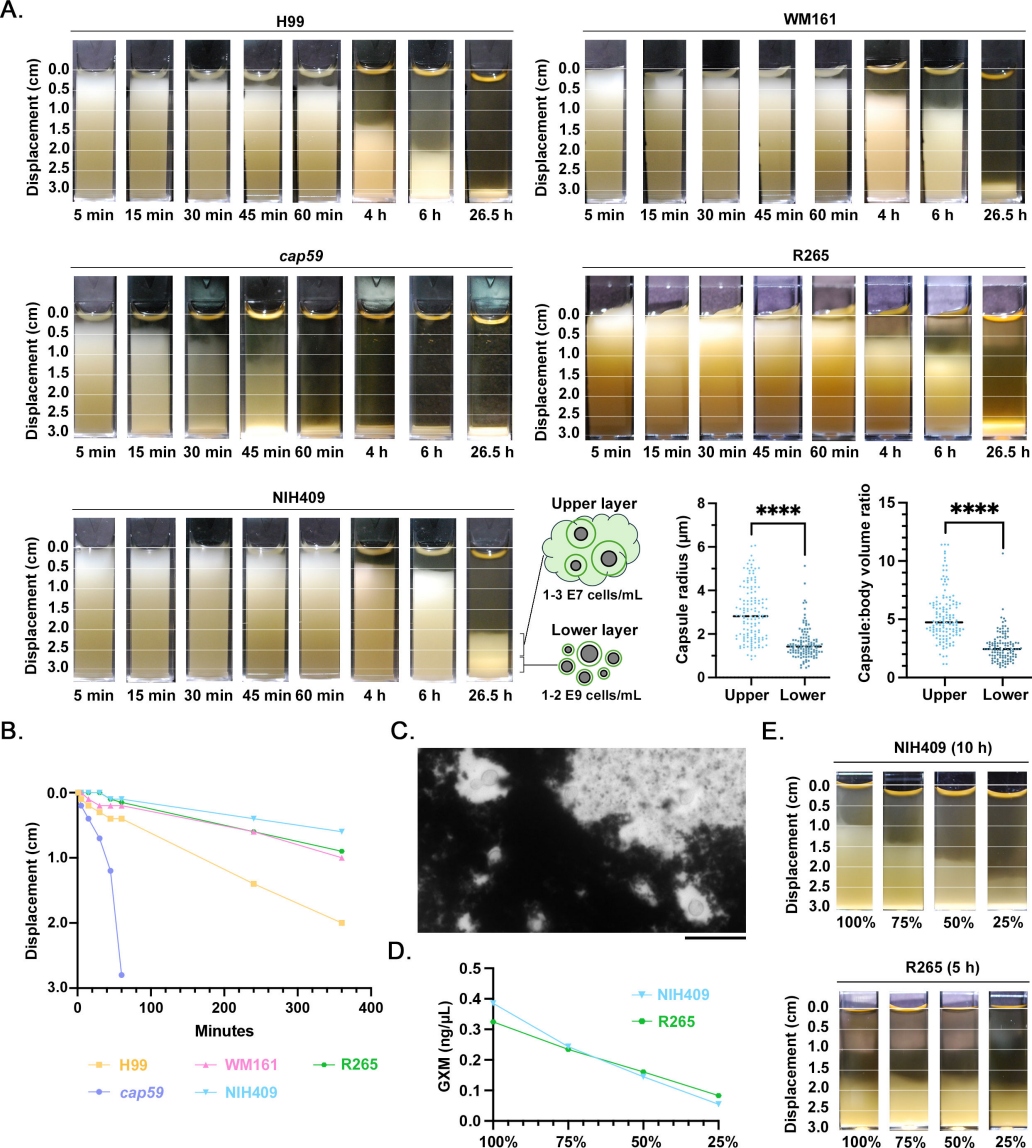
Rates of passive settling of *C. neoformans* and *C. gattii* are strain specific. *C. neoformans* strains H99 and *cap59* and *C. gattii* strains WM161, NIH409, and R265 were grown overnight in liquid YPD culture, resuspended in cuvettes, and allowed to passively settle, while photographs were taken at intervals between 5 min and 6 h, and then again at 26.5 h. Results represent two independent experiments. (**A**) The *cap59* acapsular mutant settled most rapidly, with all cells settled after 60 min. The rate of settling of *cap59* was nonlinear, with settling between 45 and 60 min occurring more rapidly than settling between 5 and 45 min. Strain H99 settled faster than strains WM161, NIH409, and R265. Strain 409 exhibited two distinct layers, even when fully settled; samples were collected from these upper and lower layers for further evaluation. The cells in the upper layer were approximately 100× more concentrated than cells in the lower layer. Cells from the upper layer had significantly larger capsule radii (*P* < 0.0001) and capsule:body volume ratio (*P* < 0.0001) compared to cells from the lower layer. We propose that formation of this upper layer after passive settling of strain NIH409 is the result of more buoyant cells in combination with a higher proportion of extracellular polysaccharide material. (**B**) Based on linear regression, the rate of passive settling was significantly different between strains (*P* < 0.0001). (**C**) Light microscopy of the upper layer of a settled strain NIH409 culture reveals copious extracellular material that induces clumping of India ink. Scale = 20 µm. (**D**) To evaluate the influence of diluting extracellular components on the rate of settling, we serially diluted stationary phase R265 and NIH409 cultures and then supplemented backwashed cells, effectively diluting out extracellular contents of the culture while keeping the cell count constant. The experimental design is depicted in more detail in Fig. S1. GXM (ng/µL) was measured in each sample by capture ELISA, demonstrating that there were no differences in the amount of GXM between strains and confirming that our methods resulted in a serial dilution of GXM. (**E**) We monitored the rate of settling of each serial dilution over a period of 10 h. Because R265 and NIH409 settle at different rates, different timepoints (5 h and 10 h, respectively) are depicted. Diluting the extracellular contents directly influenced displacement in strain NIH409 but not in strain R265. These findings suggest that varying the concentration of GXM does not uniformly affect the rate of settling, and there are strain-specific differences between NIH409 and R265.

We next assessed the rate of cryptococcal settling through water in the absence of a halocline using cell cultures that were allowed to passively settle over a period of 6 h, with an additional timepoint taken at 26.5 h (Fig. 5). The acapsular mutant *cap59* settled completely by 60 min. Cells of all encapsulated strains were incompletely settled by 6 h, with all three *C. gattii* strains settling slower than H99 (Fig. 5A). Upon observation after 26.5 h, all strains had settled, but WM161 and NIH409 still demonstrated two distinct layers, with a translucent upper layer and an opaque lower layer; this finding was much more prominent for strain NIH409. The upper layer contained approximately 100-fold fewer cells than the lower layer, and cells in the upper layer had significantly higher capsule radii (*P* < 0.0001) and capsule:body volume ratios (*P* < 0.0001) than cells from the lower layer (Fig. 5A). In addition, we microscopically visualized a large amount of acellular material that induced clumping of India ink (Fig. 5C). Rates of passive settling, as determined by the slope from 5 min to 6 h using simple linear regression, were significantly different between strains (*P* < 0.0001, *F* = 48.49; Fig. 5B). Given the lower cell density of the upper layer, we hypothesized that extracellular components were involved in contributing the bulk of this macroscopically visible volume. To evaluate the effect of the concentration of extracellular components of the culture media, we prepared a serial dilution of stationary phase cultures of strains NIH409 and R265 and then, to keep the cell count constant, supplemented cells that had been washed in fresh YPD media to remove extracellular contents (Fig. S1). We used GXM ELISA to confirm that this dilution method produced a linear decrease in GXM concentration and found that there were no differences in GXM content between the two strains (Fig. 5D). We then monitored the degree of settling over a period of 10 h. Diluting the extracellular components of the culture proportionally affected the rate of settling of strain NIH409 but not of strain R265 (Fig. 5E). These findings suggest that varying the concentration of GXM does not uniformly affect cell buoyancy, and there are strain-specific differences between NIH409 and R265.

### Passive settling results in enrichment of GXM content in the upper layer of culture material

**Fig 6:**
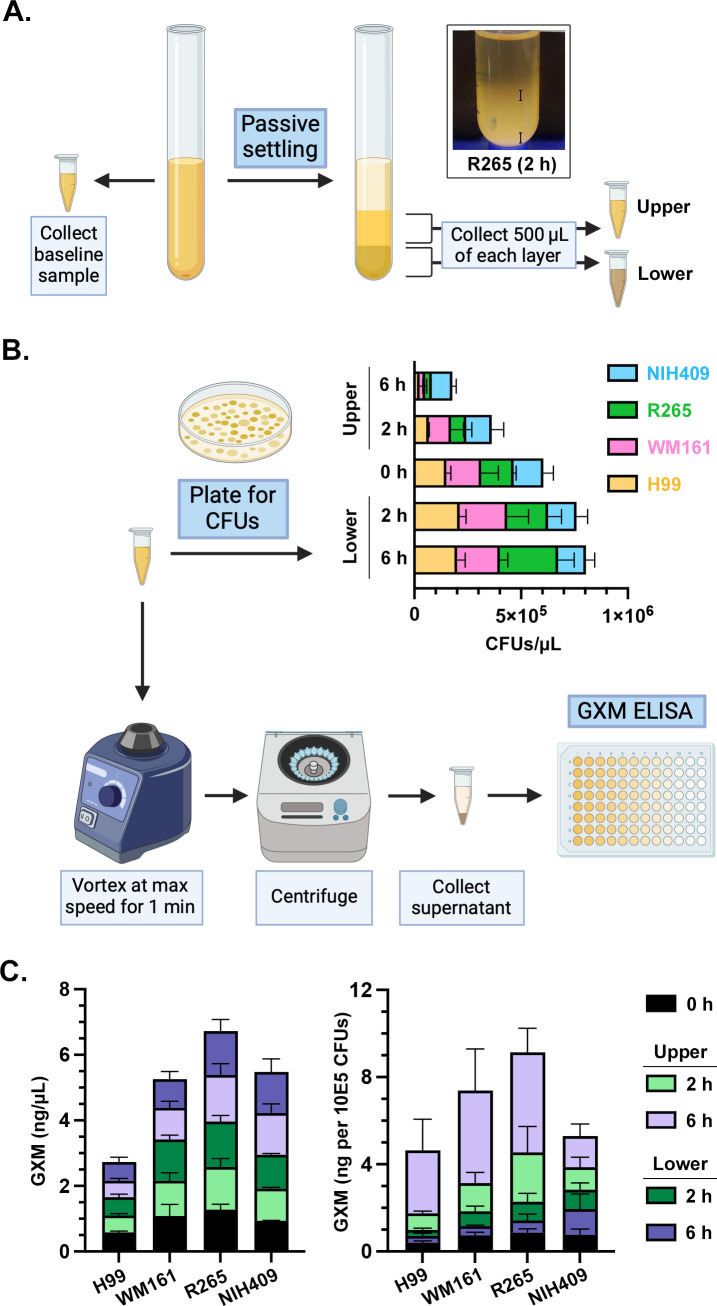
Passive settling results in the enrichment of GXM content in the upper layer of culture material. (**A**) To assess the ability of passive settling to concentrate GXM in the upper fraction, we cultured cells of strains H99, NIH409, R265, and WM161 and allowed aliquots to passively settle for 2 or 6 h. We then slowly collected 500 µL from the upper and lower regions of each sample, as shown in the representative image of strain R265. (**B**) Each sample was plated at a 1:100,000 dilution on solid YPD agar, incubated at 30°C for 2 d, after which CFUs were counted. Each sample was also vortexed at maximum speed for 1 min, centrifuged to pellet the cells, and supernatant was collected for GXM ELISA. On two-way ANOVA, CFUs varied significantly with time (*P* = 0.0014) and varied significantly with strain (*P* = 0.0005). (**C**) There was no effect of time on the concentration of GXM (ng/µL; *P* = 0.5666), suggesting that GXM remains suspended rather than settling over this timespan, but there was a significant effect of strain (*P* < 0.0001). When GXM is normalized to the cell count, there is a significant effect of time on GXM content (*P* = 0.0135), consistent with the enrichment of GXM within the upper fractions over time.

In a preliminary experiment, we found that after passive settling, the upper layer of NIH409 cultures contained a significantly higher concentration of sugars with a free-reducing group using a PSA assay (Fig. S3). We therefore sought to evaluate if GXM yield can be enhanced by allowing cultures to passively settle before collection of the upper layer. We cultured cells of four encapsulated strains and allowed cells to passively settle for 2 or 6 h before slowly collecting samples from the upper and lower layers (Fig. 6A). These samples were plated for CFUs and processed for GXM ELISA (Fig. 6B). On two-way ANOVA, CFUs varied significantly with time (*P* = 0.0014), demonstrating that cells become concentrated into the lower layer as they settle; this varied significantly with strain (*P* = 0.0005; Fig. 6B). Conversely, there was no effect of time on the concentration of GXM (*P* = 0.5666), suggesting that GXM remains suspended (Fig. 6C). There was a significant overall effect of strain on GXM content between layers (*P* < 0.0001). Given that cells settled over time while GXM remained suspended, we saw a significant effect of time on GXM content (*P* = 0.0135) when GXM was normalized to the cell count. These findings are consistent with the enrichment of GXM in the upper fraction.

### Extracellular polysaccharide in *C. gattii* cultures forms aggregates around cells

**Fig 7:**
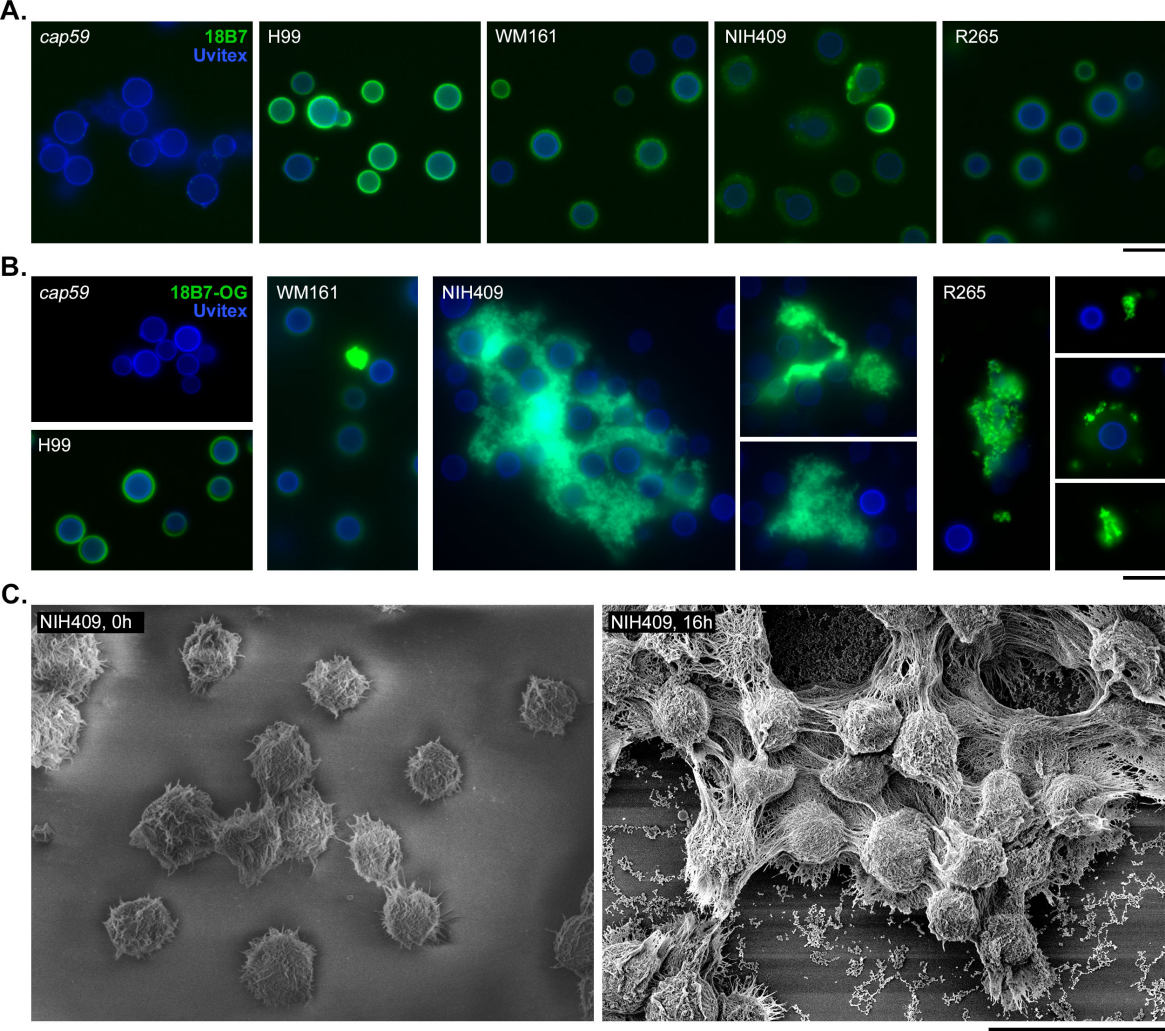
Polysaccharide in the cell supernatant forms aggregates in strains of *C. gattii*. (**A**) Immunofluorescence imaging was performed on YPD cultures of strains H99, *cap59*, WM161, and NIH409 using 18B7 anti-capsular murine mAb (green) and Uvitex 2B to stain cell wall chitin (blue). Cells were washed between incubation steps per standard immunofluorescence imaging protocols. The fluorescence intensity and distribution vary between strains, with WM161 and NIH409 having more irregular and diffuse capsule margins. The acapsular *cap59* mutant is unable to export polysaccharide to the capsule and exhibits no 18B7 fluorescence. Scale = 10 µm. (**B**) Cultures of each strain were allowed to settle, and once an upper layer was visible, samples were collected (H99, 1.5 h; WM161, 20 h; NIH409, 26.5 h; R265, 26.5 h). For *cap59*, no visible upper layer forms; therefore, a sample was taken after 45 min. Immunofluorescence imaging was performed without wash steps, using 18B7 mAb direct-conjugated to Oregon Green fluorphore (18B7-OG) and Uvitex. For all three *C. gattii* strains, we observed aggregates of material with strong 18B7 fluorescence, suggesting that they are heavily composed of polysaccharides; these varied in size by strain, with NIH409 having the largest aggregates with intricate branched structures and large clumps of polysaccharide entrapping cells. Furthermore, surrounding cells had absent, dim, or irregular binding of 18B7, suggesting that an abundance of free extracellular polysaccharide can sequester antibody. Scale = 10 µm. (**C**) SEM images of strain NIH409 following 0 and 16 h of passive settling. Scale = 10 µm.

We used immunofluorescence imaging to visualize cells at baseline and after passive settling, using mAb IGg1 18B7 to visualize the capsule and Uvitex 2B to stain cell wall chitin. When washes were performed between incubation steps, cells of strains WM161 and NIH409 had more irregular and diffuse capsule margins compared to strain H99 (Fig. 7A). The *C. neoformans* acapsular mutant, *cap59*, has impaired polysaccharide export and, thus, exhibits no capsule fluorescence. When immunofluorescence imaging was performed without wash steps on material collected from these upper layers, we observed 18B7-positive extracellular aggregates and branched structures in all three *C. gattii* strains (Fig. 7B), with varying size and complexity; strains NIH409 exhibited raft-like structures which entrapped cells. Furthermore, a subset of cells in proximity to these aggregates had absent, dim, or irregular capsular binding of 18B7. We further examined these structures in NIH409 with SEM, which revealed complex branched structures extending between and entrapping cells (Fig. 7C).

### Strain differences in baseline lipid content and lipid contribution to buoyancy

**Fig 8:**
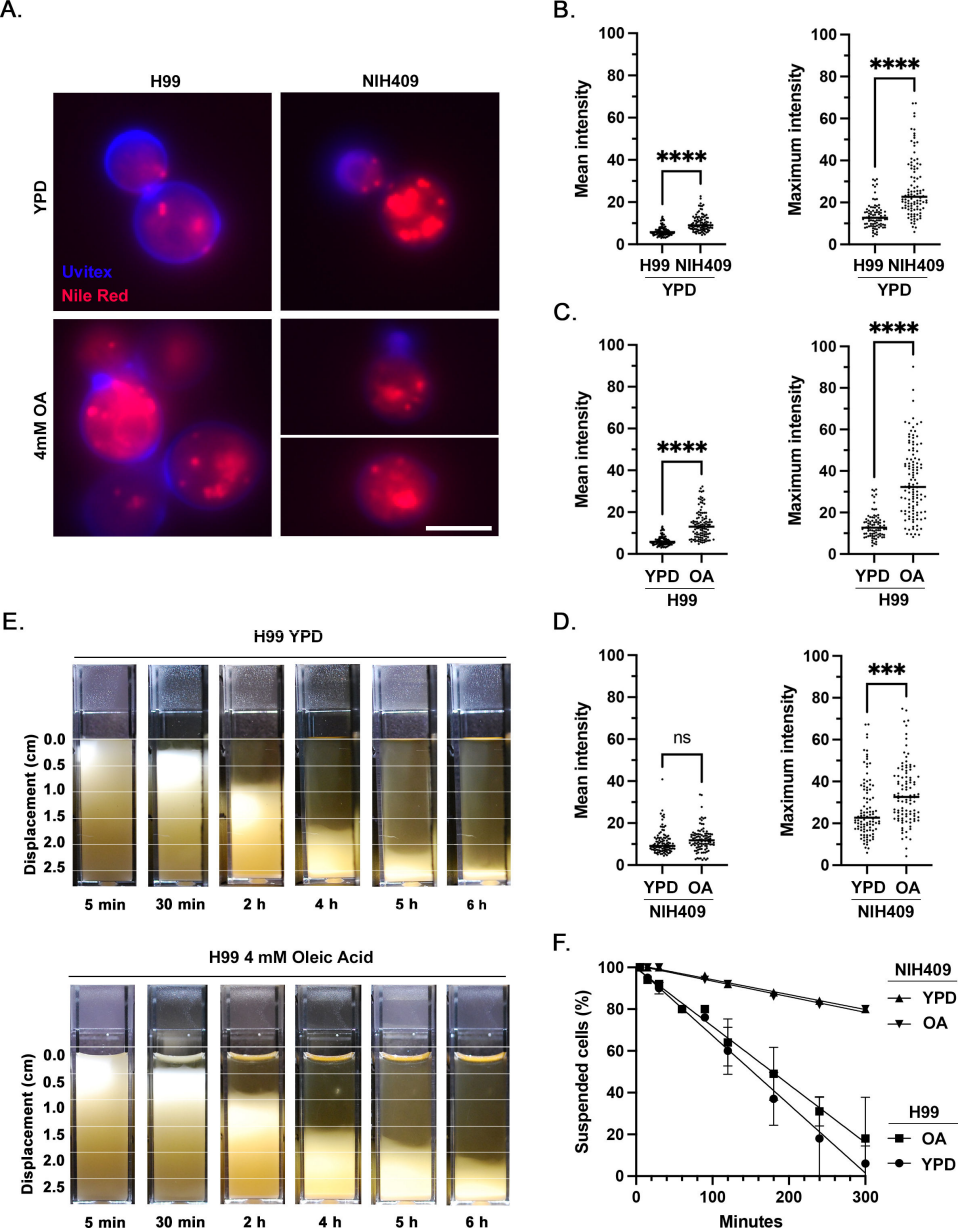
Baseline lipid content and effect of oleic acid supplementation on intracellular lipid accumulation and cell buoyancy in *C. neoformans* strain H99 and *C. gattii* NIH409. (**A**) Fluorescence microscopy images representative of relative fluorescence of neutral lipid (Nile Red, red) and cell wall chitin (Uvitex, blue) in strains H99 and NIH409, with and without oleic acid supplementation. Scale = 5 µm. (**B**) After overnight culture in plain YPD media, strain NIH409 cells had a higher baseline mean and maximum fluorescence intensity of neutral lipid compared to strain H99. (**C and D**) After overnight culture in YPD media supplemented with 4 mM oleic acid, cells of strain H99 exhibited higher mean and maximum fluorescence intensity of neutral lipid compared to baseline, while cells of strain NIH409 exhibited no significant change in mean fluorescence intensity but had a higher maximum fluorescence intensity compared to baseline. (**E**) H99 cells were grown overnight in plain YPD media or YPD media supplemented with 4 mM oleic acid and then transferred to cuvettes and allowed to passively settle. Data represent the results of two independent experiments. (**F**) Rate of cell settling by simple linear regression. Strain NIH409 cultures settled significantly slower than strain H99 cultures (*P* < 0.0001), while culture conditions did not result in a statistically significant difference in the rate of settling for strain NIH409 (*P* = 0.4539) or strain H99 (*P* = 0.0700) at the *α* = 0.05 level.

Having found strain-specific differences in the rate of settling, we sought to evaluate other factors which may contribute to cell buoyancy. First, we assessed the baseline lipid content of strains H99 and NIH409 using Nile Red stain for neutral lipid (Fig. 8A). After overnight culture in YPD media, strain NIH409 cells had significantly higher mean (*P* < 0.0001) and maximum (*P* < 0.0001) fluorescence intensity of neutral lipid compared to strain H99 (Fig. 8B).

Supplementing YPD media with 4 mM oleic acid during overnight culture of strain H99 was sufficient to induce lipid droplet formation, corresponding to higher mean (*P* < 0.0001) and maximum (*P* < 0.0001) fluorescence intensity of neutral lipid compared to baseline (Fig. 8C). Supplementing YPD media with 4 mM oleic acid during overnight culture of strain NIH409 resulted in a higher maximum Nile Red fluorescence intensity compared to cells grown in plain media (*P* = 0.0002) but did not significantly affect mean fluorescent intensity (*P* = 0.1230; Fig. 8D). To assess whether inducing an increase in lipid content via oleic acid supplementation would affect the rate of cell settling, we cultured cells of strains NIH409 and H99 in YPD with and without oleic acid, then transferred cells to cuvettes and allowed them to passively settle. There was no significant difference in the rate of cell settling for NIH409 cells between growth conditions (*P* = 0.4539). Although H99 cells cultured in oleic-acid supplemented media appeared visually to settle slightly slower than cells in plain media at certain timepoints, differences in overall rate of cell settling between culture conditions did not reach statistical significance at the *α* = 0.05 level (*P* = 0.0700; Fig. 8F).

### Proposed model of interaction of cryptococci with natural aqueous environments

**Fig 9:**
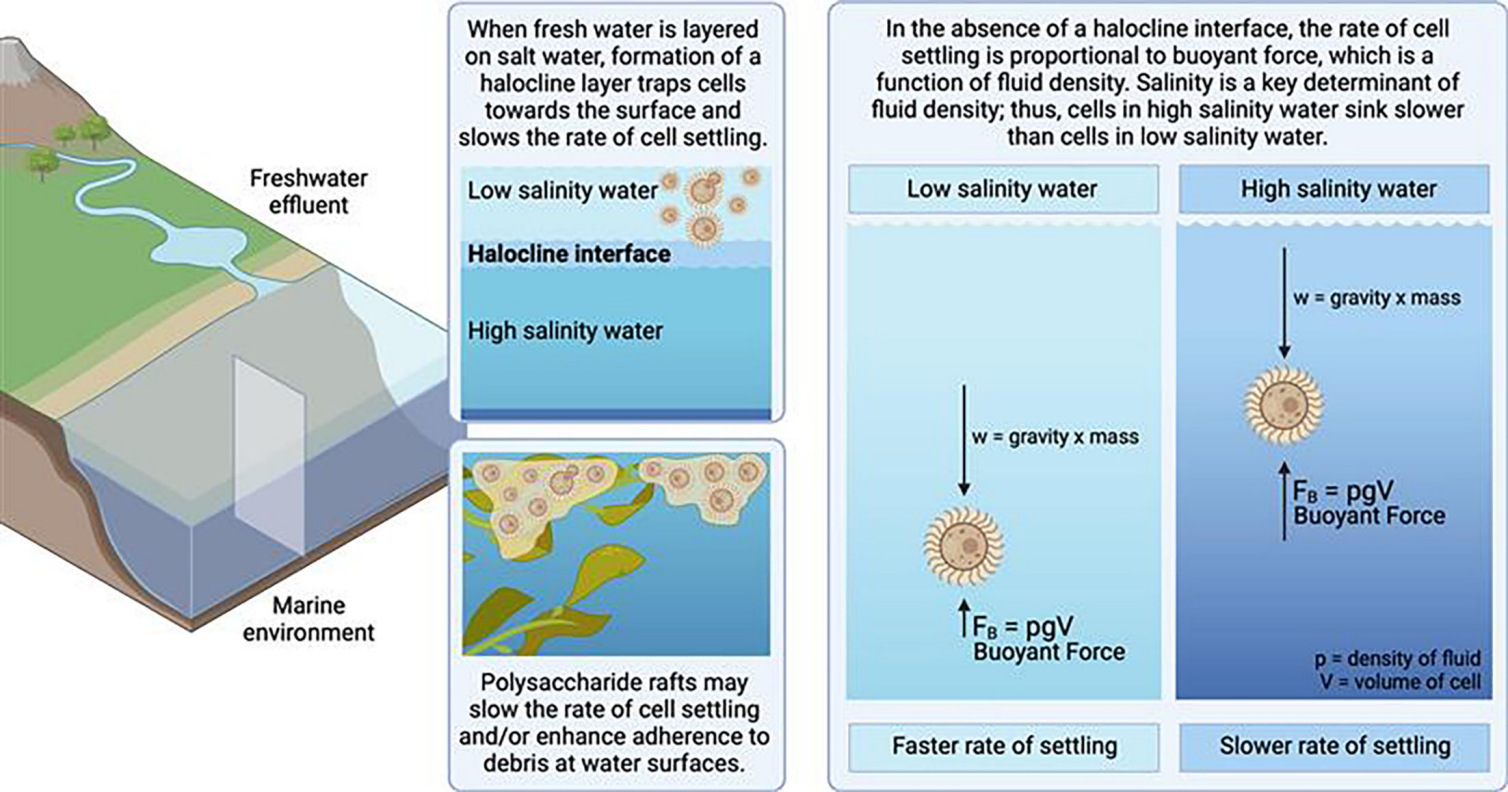
Proposed model of interaction of cryptococci in natural aqueous environments. We propose that cryptococcal cells residing in terrestrial reservoirs, such as trees and soil, can be carried by freshwater effluents into marine environments. The layering of fresh water over salt water results in the formation of a halocline interface, suspending cryptococci closer to the water surface and slowing the rate of cell settling. In the absence of a halocline, the rate of cell settling is a function of mass, gravity, and the salinity of the water, with higher salinity water contributing more buoyant force. Thus, marine environments would be expected to slow the rate of cell settling and may enable long-range cryptococcal transport via ocean currents. Polysaccharide rafts, being less dense than cryptococcal cells, may also slow the settling rate of entrapped cells and/or enhance adherence to debris at water surfaces, facilitating biofilm formation. Created with BioRender.com.

We propose that cryptococcal cells from terrestrial reservoirs can be carried by freshwater effluents into marine environments, where layering of fresh water over salt water results in the formation of a halocline interface that enhances cryptococcal buoyancy and prolongs their persistence close to the water surface (Fig. 9). In the absence of a halocline, the rate of cell settling is a function of the cell’s gravity and the salinity of the water, with higher salinity water contributing more buoyant force. The formation of polysaccharide rafts would further prolong cell settling and enhance adherence to debris or biofilm formation.

## DISCUSSION

The ecological niche for pathogenic cryptococci is thought to be primarily land based, and inhalation of dry aerosolized spores or desiccated yeasts from terrestrial reservoirs is the primary documented mode of infection. However, terrestrial reservoirs for cryptococci are also exposed to rain, agricultural runoff, and wind, and cells could thus be carried to aquatic environments. Wildfire smoke, for instance, has been shown to transport viable microbes, including fungi (55–58). Natural infection of marine mammals with *C. gattii* raises the potential of respiratory exposure to cryptococci through inhalation of cells from the air-water interface, including cells deposited by wind or suspended in water currents. Because marine mammals are intermittent breathers that hold inspired air in their lungs while underwater, their breathing pattern begins with rapid, forceful exhalation of spent air shortly after breaching the surface (59–61). Dolphins, for instance, expel up to 130 L/s of air (61) at speeds of over 20 m/s, aerosolizing surface water in the process (62), before rapidly inhaling a mixture of air and spray. This presents an opportunity for pathogens in the water column to become aerosolized and inhaled, and suggests that liquid droplets and wet aerosol deserve further consideration as modes of exposure.

Few studies have evaluated cellular structures and variables affecting the aqueous transport of cryptococci. Vij et al. (36) demonstrated that cryptococci with large capsules had lower cellular density and cells without capsules had higher density, suggesting that the capsule could confer buoyancy and facilitate aqueous transport (36). Capsular polysaccharides are highly hydrophilic and the capsule is highly intercalated with water, forming a hydrated shell around the cell body (63). The *C. neoformans* capsule has negatively charged glucuronic acid groups that bind divalent cations (54) and contribute to the repulsion of cells (64). Conversely, acapsular cells are notoriously clumpy when examined microscopically (64), a property that would accelerate settling (65) and which we observed was enhanced in the presence of seawater, with macroscopic clumps of *cap59* cells adhering to the cuvette. The outer surface of the capsule in some strains is also hydrophobic (66), which may keep encapsulated cryptococci spaced apart as they settle through water, further contributing to cell suspension.

Multiple findings in the present study confirm the role of the capsule in enhancing buoyancy. The rate of passive settling of cryptococcal cells in liquid culture varied significantly by strain and correlated with strain-specific cell densities, with cells of the *cap59* acapsular mutant being most dense and sinking most rapidly, followed by strains H99, WM161, R265, and finally NIH409. Strains with lower cell density also had higher capsule:body volume ratios. On immunofluorescence imaging, capsules of *C. gattii* strains WM161 and NIH409 were also more diffuse in appearance than capsules of H99 grown under the same conditions; differences in the structure of the capsule could also affect cell settling.

We also observed that all three *C. gattii* strains manifested significantly larger capsules after 3 d incubation in PBS. Capsule growth is a response to cellular stress, such as in nutrient-poor environments; the ability to build capsule more readily in aquatic environments could be an additional strain-specific strategy to modulate buoyancy. Interestingly, *C. gattii* strain R265, which is the strain principally responsible for the outbreaks of cryptococcosis in the PNW and Vancouver Island, also exhibited a larger capsule after 3 d incubation in SW, a finding that was not present in any other strains studied. To differentiate capsule growth from acute, reversible physicochemical expansion of the capsular gel in response to hypotonic solution, as described by Jacobson et al. (48), we evaluated the capsule size of strain R265 after dialysis in a series of salt solutions. We found no significant differences between conditions under these acute conditions, suggesting that the previously observed increases in capsule size were associated with capsular growth over the 3 d incubation period. Interestingly, R265 was the only *C. gattii* strain for which capsule size increased during SW incubation. In most strains, capsule growth was not significantly induced by incubation in SW, supporting our hypothesis that capsule induction is not the sole mechanism by which cryptococci modulate buoyancy in natural environments. We therefore sought to evaluate other factors involved in cryptococcal cell buoyancy.

Because lipids are a common intracellular macromolecule and are less dense than water, we hypothesized that lipid content also influences cell density. Of the four encapsulated strains in this study, we evaluated baseline lipid content in the least dense (NIH409) and most dense (H99) strains and found that strain NIH409 had a higher baseline lipid content than strain H99. We then supplemented the growth media for both strains with oleic acid to induce lipid uptake. Lipid content, as measured by the fluorescent intensity of Nile Red stain for neutral lipid, increased in both strains but was more pronounced in H99 than in NIH409, potentially because NIH409’s higher baseline lipid left less room for further uptake. We then evaluated the rate of passive settling in both strains, with and without supplementation of oleic acid. Culture conditions did not result in a statistically significant difference in the rate of settling for either strain, although visually, cells of strain H99 grown in lipid-supplemented media appeared to settle slower than cells grown in plain YPD media. Further studies could evaluate the role of lipid content in promoting buoyancy.

We also sought to evaluate factors extrinsic to the cell that could contribute to buoyancy. In this study, at various times of passive settling, all encapsulated stains developed a translucent upper region; this was not observed for the *cap59* acapsular mutant, consistent with prior work suggesting that the *CAP59* gene is essential for polysaccharide export (39). This finding was most pronounced for strain NIH409, in which a large distinct upper layer was visible after over 24 h. On microscopic evaluation, this layer of NIH409 contained copious acellular material interspersed with cells with a significantly higher capsule:body volume ratio than cells in the lower layer; the cell density of the upper layer was also 100-fold lower than that of the lower layer. SEM images of this upper layer revealed strands of material coating and connecting cells and forming complex structures. Immunofluorescence imaging and GXM ELISA results support that this material is largely composed of GXM polysaccharide, which is also the principal component of the cryptococcal capsule. In the context of these findings, we hypothesize that polysaccharide secreted or shed during growth of encapsulated strains could form aggregates that then entrap cells and, being less dense than the cells themselves, act as a raft to enhance buoyancy. *C. neoformans* and *C. gattii* secrete EPS during culture and infection (67, 68). Nimrichter et al. (54) further identified that a viscous, jelly-like mat of entangled GXM fibers was present on filter paper after ultrafiltration of *C. neoformans* culture (54). EPS from *C. laurentii* was also reported to facilitate and stabilize oil-water emulsions and to increase viscosity and drag, both of which would slow the rate of cell settling (69). Interestingly, however, despite the marked differences in the rate of settling between strains in the present study, baseline GXM concentrations did not differ significantly between strains. Furthermore, in a direct comparison of strains NIH409 and R265 with both strains having similar GXM concentrations, diluting the extracellular components of a stationary phase culture directly impacted the rate of settling of NIH409 but not R265. Thus, varying the concentration of GXM does not uniformly affect the rate of settling. Given the strain-specific variations in the sizes of GXM aggregates observed on immunofluorescence imaging, we hypothesize that buoyancy is affected not strictly by the amount of GXM produced but rather by the size and structure of the GXM aggregates. Larger aggregates would be expected to more easily entrap cells and may, therefore, have a relatively larger impact on cell flotation compared to GXM that forms small/no aggregates. Thus, the discovery of polysaccharide aggregates suggests a new role for EPS in promoting aqueous transport and suggests new avenues for research into factors influencing the structure and composition of these rafts. Furthermore, if present during growth of these cells in nature, GXM aggregates may participate in adherence of cells to debris and/or biofilm formation, both of which could enhance persistence in aqueous environments.

Different laboratory methods of EPS isolation have varying effects on polysaccharide organization, structure, and aggregation (67, 70, 71). In this study, PSA and GXM ELISA results suggest that allowing a culture to passively settle until a translucent upper layer can be collected enriched the sample for EPS compared to direct sampling of a stationary phase culture. This method may supplement existing EPS collection techniques. Strain-specific differences in settling time should be considered when determining the optimal time to sample this layer and may reflect differences in amount, composition, or structure of EPS. High concentrations of EPS also appeared to inhibit binding of 18B7 mAb to neary cells. The role of EPS in sequestering cells from antibodies may be an immune evasion mechanism, given the importance of antibody-mediated opsonization in the response to cryptococcal infection. Aggregates of polysaccharide and cells have been described during *in vitro* infection of macrophages with *C. neoformans* or *C. gattii* (68) and in the context of cryptococcal biofilm formation (72–74). Our methods also preserved macromolecular structures, allowing visualization of relationships between polysaccharide aggregates and entrapped cells, which may be applicable to future studies of biofilm formation.

Potential mechanisms for cryptococcal cells to move from land to sea include cells being carried by wind and deposited onto the water surface, or cells being washed from soil by rain, runoff, or other freshwater effluents. To test the hypothesis that cryptococci suspended in freshwater could be carried to the sea and then persist near the water surface, we experimentally replicated the ecological phenomenon of halocline formation, in which low salinity water forms a stable layer above seawater, such as in estuaries and caves. In nature, particles traverse the halocline as a function of their density and can become suspended at this interface, creating a unique composition of nutrients, debris, and microbes (75). In the presence of a halocline created at environmentally relevant salinities, cells were initially suspended near the water surface, with all four encapsulated strains remaining within 1 cm of the water surface for over 60 min. Conversely, in the absence of a halocline, cells grown under identical culture conditions rapidly sank. The halocline was also observed to suspend cells of the *cap59* mutant near the water surface (although for a shorter time), confirming that this initial effect is independent of the presence of the capsule. In a test of the dynamic stability of the halocline layer, horizontal agitation did not result in halocline disruption until speeds exceeded 100 rpm, at which time the water surface became turbulent; this suggests that cryptococci could possibly remain suspended while carried by natural water currents. Because higher-density fluids confer more buoyant force, cells also settled slower in higher salinity media than in lower salinity media. When overall salinity was kept constant, the presence of the halocline layer significantly slowed the rate of settling. Thus, both salinity and the presence of the halocline layer are additional extrinsic factors that influence the rate of settling of cryptococcal cells in water.

In this study, we observed strain-specific differences in cell density that directly correlated with the rate of cell settling and identified factors that contribute to buoyancy, including capsule size, polysaccharide production, salinity of the surrounding media, and interaction with the halocline. We also observed that *C. gattii* strains appeared to respond to incubation in water by inducing capsular growth, a feature absent in *C. neoformans*. By increasing persistence near the water surface via these various mechanisms, cryptococci have multiple strategies to increase the likelihood of transport by waves to new environmental niches, encounter debris upon which to form biofilms, and encounter susceptible hosts.

## SUPPLEMENTAL MATERIAL

Supplemental material msphere.00848-24-s0001.docx Tables S1 and S2, Fig. S1-S3, and Video S1 legend.

Video S1 msphere.00848-24-s0002.mp4 Halocline persistence is dynamic.

ASM does not own the copyrights to Supplemental Material that may be linked to, or accessed through, an article. The authors have granted ASM a non-exclusive, world-wide license to publish the Supplemental Material files. Please contact the corresponding author directly for reuse.
