# Supplementary material for: The buoyancy of cryptococcal cells and its implications for transport and persistence of Cryptococcus in aqueous environments
Source: mSphere. 2024 Nov 27;9(12):e00848-24. doi: 10.1128/msphere.00848-24 (PMC11656806; doi:10.1128/msphere.00848-24)
Supplement: Supplemental material — Tables S1 and S2, Fig. S1-S3, and Video S1 legend. [file msphere.00848-24-s0001.docx]

**Supplemental Table 1: Analysis of seawater elements by Inductively Coupled Plasma Optical Emission Spectroscopy (ICP-OES)**

| **Element** | **Name** | **Nutri Live Seawater** | **Pacific Ocean Seawater** | **Setpoint** | **Unit** |
| --- | --- | --- | --- | --- | --- |
| **Sal** | Salinity | 35.148 | 35.084 | 35 | PSU |
| **Cl** | Chloride | 19813 | 19770 | 19500.00 | mg/l |
| **Na** | Sodium | 10863 | 11023 | 10000 - 11300 | mg/l |
| **Mg** | Magnesium | 1401 | 1311 | 1320 - 1500 | mg/l |
| **S** | Sulphur | 933 | 917 | 900.00 | mg/l |
| **Ca** | Calcium | 444 | 421 | 415 - 520 | mg/l |
| **K** | Potassium | 415 | 393 | 380 - 480 | mg/l |
| **Br** | Bromide | 63 | 64 | 64.00 | mg/l |
| **Sr** | Strontium | 9 | 8 | 45516.00 | mg/l |
| **B** | Boron | 5 | 5 | 4.50 | mg/l |
| **F** | Fluoride | 1.28 | 1.45 | 1.30 | mg/l |
| **PO4** | Phosphate | 0.021 | 0.113 | 0.018 - 0.07 | mg/l |
| **Si** | Silicon | 293 | 424 | 0 - 200 | µg/l |
| **Li** | Lithium | 204 | 181 | 200.00 | µg/l |
| **I** | Iodine | 23 | 26 | 30 - 90 | µg/l |
| **Ba** | Barium | 11 | 13 | 0 - 10 | µg/l |
| **Mo** | Molybdenum | 8 | 7 | 12.00 | µg/l |
| **P** | Phosphorus | 7 | 37 | 45466.00 | µg/l |
| **Al** | Aluminium | 0 | 0 | 0 - 60 | µg/l |
| **Sb** | Antimony | 0 | 0 | 0.00 | µg/l |
| **As** | Arsenic | 0 | 0 | 0.00 | µg/l |
| **Be** | Beryllium | 0 | 0 | 0.00 | µg/l |
| **Bi** | Bismuth | 0 | 0 | 0.00 | µg/l |
| **Pb** | Lead | 0 | 0 | 0.00 | µg/l |
| **Cd** | Cadmium | 0 | 0 | 0.00 | µg/l |
| **Cs** | Caesium | 0 | 0 | 0.00 | µg/l |
| **Ga** | Gallium | 0 | 0 | 0.00 | µg/l |
| **In** | Indium | 0 | 0 | 0.00 | µg/l |
| **Co** | Cobalt | 0 | 0 | 0.00 | µg/l |
| **Cu** | Copper | 0 | 0 | 0.00 | µg/l |
| **La** | Lanthanum | 0 | 0 | 0.00 | µg/l |
| **Hg** | Mercury | 0 | 0 | 0.00 | µg/l |
| **Sc** | Scandium | 0 | 0 | 0.00 | µg/l |
| **Ag** | Silver | 0 | 0 | 0.10 | µg/l |
| **Se** | Selenium | 0 | 0 | 0.00 | µg/l |
| **Te** | Tellurium | 0 | 0 | 0.00 | µg/l |
| **Ti** | Titanium | 0 | 0 | 0.00 | µg/l |
| **W** | Tungsten | 0 | 0 | 0.00 | µg/l |
| **Sn** | Tin | 0 | 0 | 0.00 | µg/l |
| **Ni** | Nickel | 0 | 0 | 5.00 | µg/l |
| **V** | Vanadium | 0 | 0 | 0 - 3 | µg/l |
| **Zn** | Zinc | 0 | 0 | 0 - 5 | µg/l |
| **Mn** | Manganese | 0 | 0 | 0 - 3 | µg/l |
| **Cr** | Chromium | 0 | 0 | 0.00 | µg/l |
| **Fe** | Iron | 0 | 0 | 0.00 | µg/l |

**Supplemental Table 2: Concentrations and specific gravity of sodium chloride solutions**

| **NaCl Molarity (M)** | **Specific gravity** |
| --- | --- |
| 0.026 | 1.002 |
| 0.051 | 1.003 |
| 0.163 | 1.007 |
| 0.363 | 1.016 |
| 0.676 | 1.028 |
| 1.506 | 1.060 |


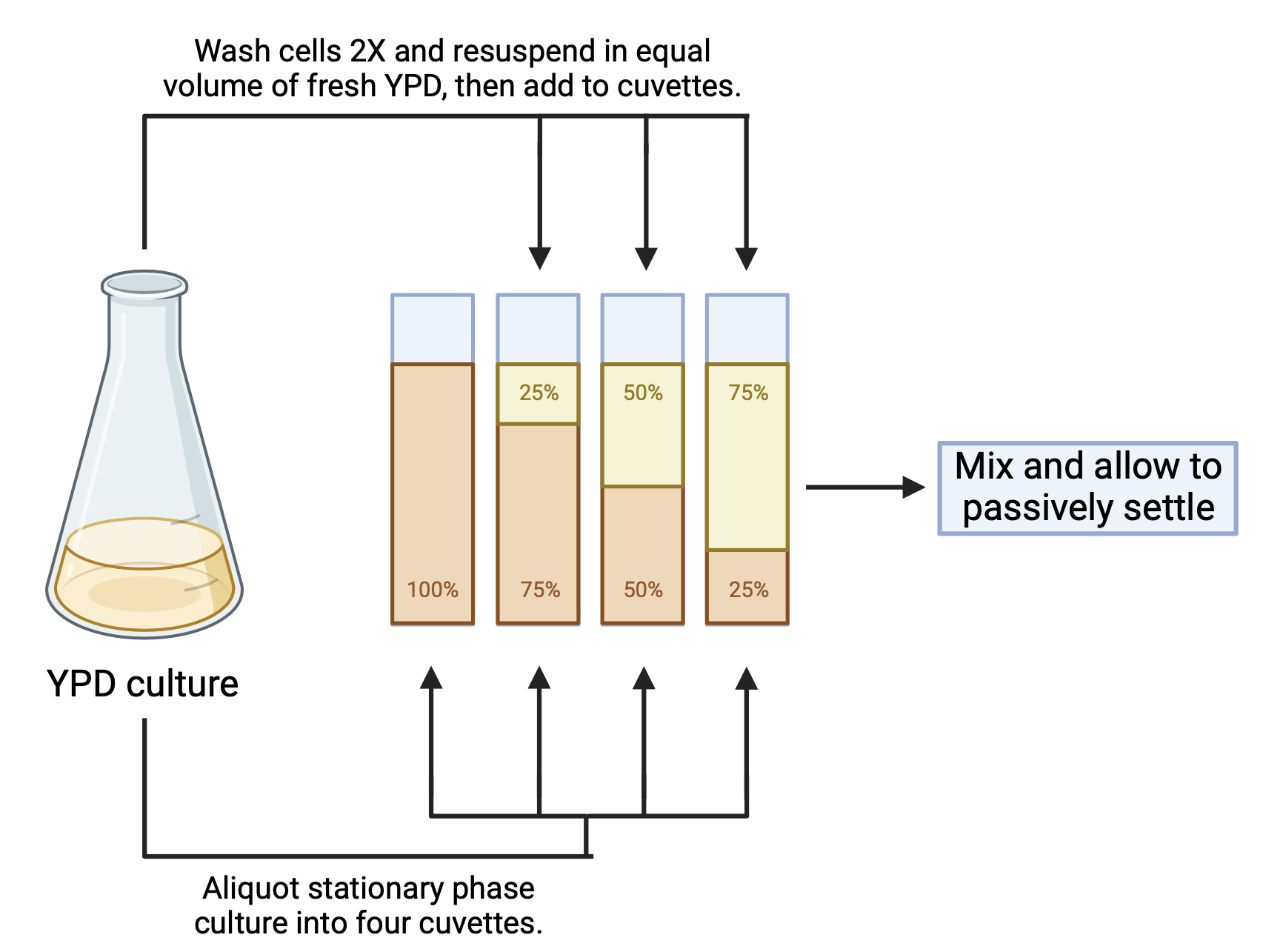


**Supplemental Figure 1. Experimental design of polysaccharide dilution curve.** To evaluate the influence of the amount of extracellular GXM on the rate of settling, stationary phase cultures of strain NIH409 and R265 were serially diluted such that 3 mL, 2.25 mL, 1.5 mL, and 0.75 mL of the original culture was supplemented with 0 mL, 0.75 mL, 1.5 mL, or 2.25 mL, respectively, with cells that had been washed cells twice and resuspended in fresh YPD media. Thus, the cell count was kept constant while the GXM content was reduced.

**
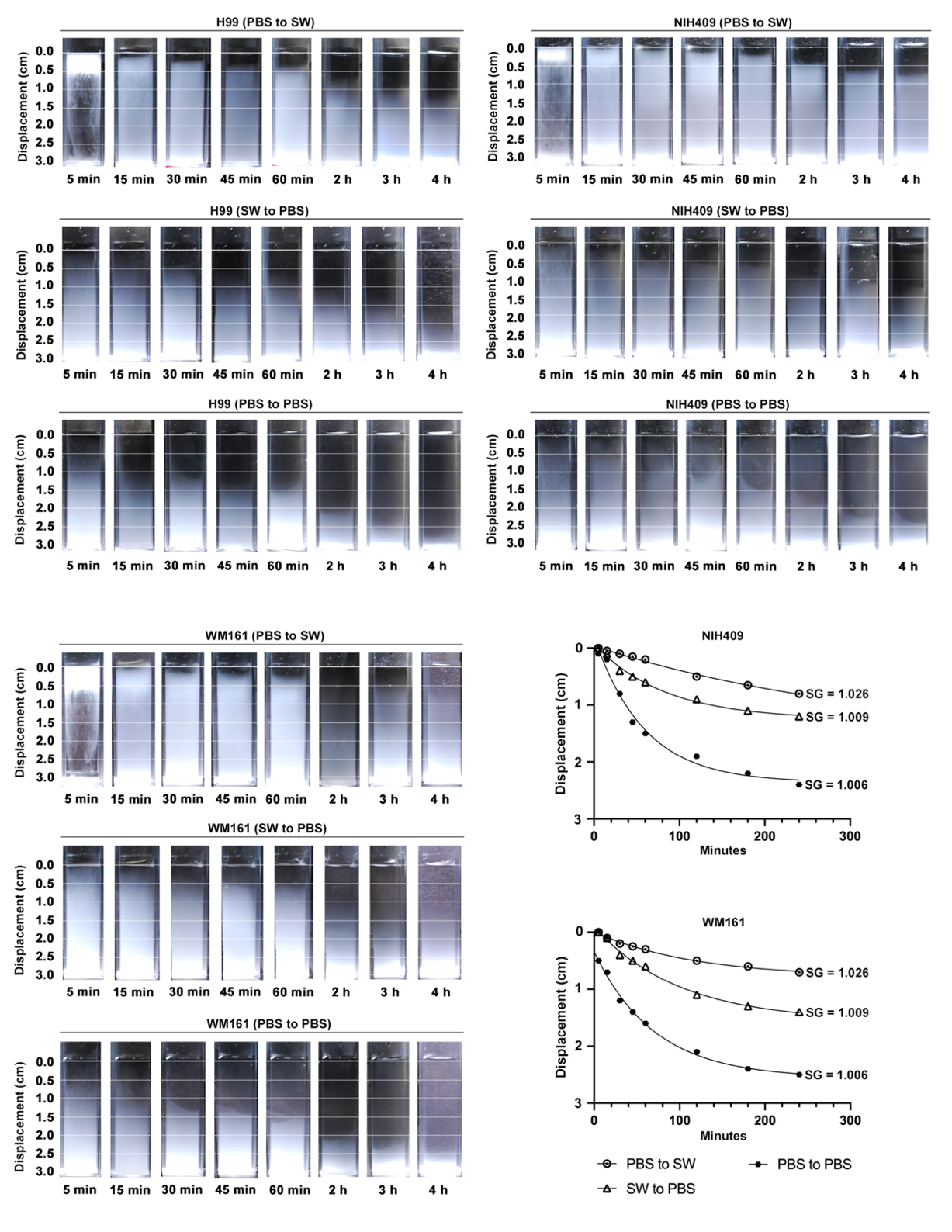
**

**Supplemental Figure 2. Salinity and halocline formation affect the rate of settling of cryptococcal strains H99, NIH409, and WM161.** Cells of strains H99, WM161, and NIH409 were suspended in 200 µL of PBS or seawater (SW) and added to the top of cuvettes containing 3 mL of either PBS or SW. Cells suspended in PBS and added to SW were initially suspended at the halocline interface and then moved out of the halocline over time. The rate of cell settling was assessed over 4 h by measuring displacement (cm) from the top of the cuvette. Media type significantly impacted the rate of cell settling (P<0.0001). Cells suspended in PBS and added to PBS (final SG=1.006) settled the fastest, while cells suspended in PBS and added to SW (final SG = 1.026) settled the slowest. This trend was conserved for all three strains. Results for strain H99 are shown in Figure 4.


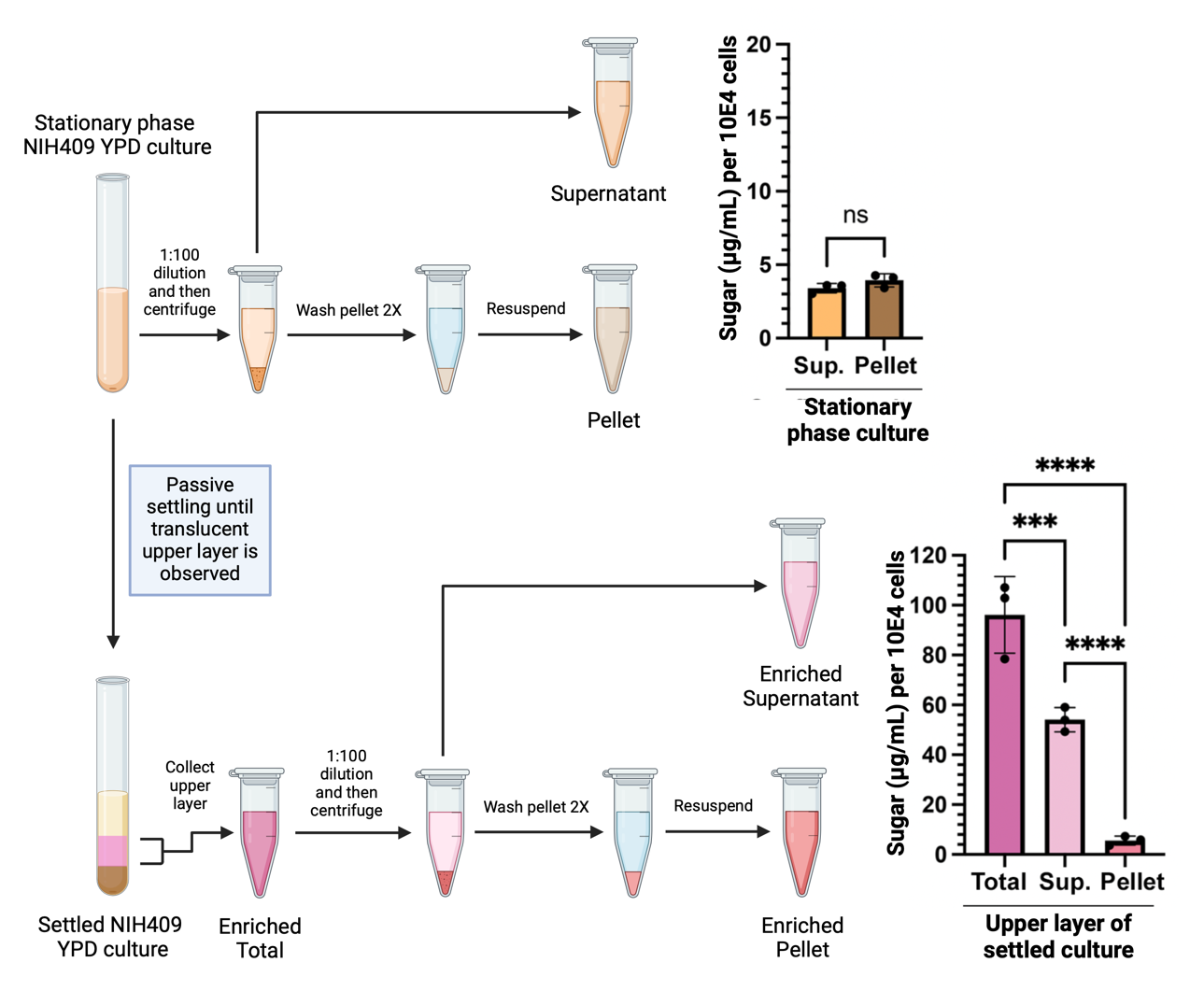


**Supplemental Figure 3:** **Total sugars are concentrated into the supernatant post-settling.** To assess the ability of passive settling to concentrate polysaccharide into a visible upper layer which can then be collected, we utilized a phenol-sulfuric assay (PSA), which quantifies sugars with a free reducing group. A 500 µL sample was taken from a stationary phase culture of *C. gattii* strain NIH409, centrifuged, and the supernatant was collected for PSA. The cell pellet was then washed twice before also being processed for PSA. Sugar concentration was then normalized to the cell count of each sample. There was no significant difference in sugar concentration between the pellet and supernatant (P>0.9999) of the stationary phase culture. Next, a stationary phase culture of strain NIH409 was allowed to passively settle for approximately 18 h at room temperature, before collection of 500 µL of the translucent upper layer; this sample was processed for PSA using the same methods described. The concentration of sugar in the supernatant collected from the post-settling sample was significantly higher than that of the original sample (P<0.0001), and significantly higher than the sugar concentration of the cell pellet (P<0.0001). These results demonstrate that sugar concentration can be enriched by allowing a stationary phase culture to passively settle until a visible layer can be sampled. Further experiments using ELISA were then performed to confirm that these findings corresponded to the enrichment of GXM polysaccharide, as described in the manuscript text.

**Supplemental Video 1: Halocline persistence is dynamic.** Cells of strain R265 were suspended in PBS dyed with phenol red, and this cell suspension was layered onto seawater in a culture flask. The flask was placed on a horizontal plate shaker and rotations per min (rpm) was incrementally increased, demonstrating that the halocline persisted at speeds of up to 100 rpm.

[See Supplemental Video File]
